# A revision of the Chinese Aulacidae (Hymenoptera, Evanioidea)

**DOI:** 10.3897/zookeys.587.7207

**Published:** 2016-05-10

**Authors:** Hua-yan Chen, Giuseppe Fabrizio Turrisi, Zai-fu Xu

**Affiliations:** 1 Department of Entomology, The Ohio State University, 1315 Kinnear Road, Columbus, Ohio 43212, U.S.A.; 2 Via Cristoforo Colombo 8, 95030, Pedara, Catania, Italy; 3 Department of Entomology, South China Agricultural University, Guangzhou 510640, P. R. China

**Keywords:** Aulacidae, Aulacus, China, keys, new species, Pristaulacus, revision, taxonomy

## Abstract

The Chinese Aulacidae are revised, keyed and illustrated for the first time. In total twenty-five species are recorded from China, included within two genera *Aulacus* Jurine, 1807 and *Pristaulacus* Kieffer, 1900, with five and twenty species respectively. Among the treated species, six are newly described for science: *Aulacus magnus* **sp. n.**, *Pristaulacus calidus* **sp. n.**, *Pristaulacus centralis* **sp. n.**, *Pristaulacus fopingi* **sp. n.**, *Pristaulacus obscurus* **sp. n.**, and *Pristaulacus pseudoiosephi* **sp. n.** Three species are newly recorded from China: *Pristaulacus excisus* Turner, 1922, *Pristaulacus iosephi* Turrisi & Madl, 2013, and *Pristaulacus rufobalteatus* Cameron, 1907.

## Introduction

Aulacidae (Evanioidea) are a small cosmopolitan family, with two extant genera, containing 247 recognized species: *Aulacus* Jurine, 1807, with 77 species, and *Pristaulacus* Kieffer, 1900, with 170 species (Smith 2001, 2005a, 2005b, 2008; Jennings and Austin 2006; Turrisi et al. 2009; Smith and Carvalho 2010; Turrisi and Konishi 2011; Turrisi and Watanabe 2011; Turrisi 2013a, 2014; Turrisi and Madl 2013; Watanabe et al. 2013; Sundukov and Lelej 2015). Both genera occur in all zoogeographic regions, except Antarctica, although *Aulacus* is not known from the Afrotropics (Kieffer 1912; Hedicke 1939; Smith 2001; Turrisi 2004; Turrisi 2006; Turrisi et al. 2009). Most species of this family occur in tropical and subtropical regions (Smith 2001; Jennings et al. 2004a, 2004b, 2004c; Turrisi et al. 2009). Aulacids are endoparasitoids of wood-boring larvae of Xiphydriidae (Hymenoptera), Buprestidae and Cerambycidae (Coleoptera) (Barriga 1990; Gauld and Hanson 1995; Smith 2001; Jennings and Austin 2004; Jennings et al. 2004a).

China is located between two zoogeographical regions, Palaearctic and Oriental, and thus includes mixed faunistic characters of both regions. However, Chinese Aulacidae are currently very poorly known (Turrisi 2007) and there have been no comprehensive revisionary attempts, although a few scattered taxonomic papers have been published since the World Aulacidae catalogue by Smith (2001) (He et al. 2002; He 2004; Turrisi 2005, 2007; Sun and Sheng 2007a, 2007b; Turrisi and Smith 2011; Sundukov and Lelej 2015). To date, only sixteen species are recorded from China, four *Aulacus* and twelve *Pristaulacus* (Table 1). This number is believed to be an underestimate, suggesting the need for extensive investigation and more research for a better knowledge of the Chinese Aulacidae (Turrisi 2007).

**Table 1.** List of the Chinese species of Aulacidae before this study, with distribution in China.

Species	Chinese distribution
*Aulacus flavigenis* Alekseev, 1986	Heilongjiang
*Aulacus schoenitzeri* Turrisi, 2005	Shaanxi
*Aulacus sinensis* He & Chen, 2007	Zhejiang
*Aulacus striatus* Jurine, 1807	Inner Mongolia
*Pristaulacus albitarsatus* Sun & Sheng, 2007	Henan
*Pristaulacus asiaticus* Turrisi & Smith, 2011	Hubei
*Pristaulacus comptipennis* Enderlein, 1912	Taiwan, Hongkong
*Pristaulacus intermedius* Uchida, 1932	Shaanxi
*Pristaulacus karinulus* Smith, 2001	Henan, Jiangsu, Taiwan
*Pristaulacus longicornis* Kieffer, 1911	China (unknown whether Palaearctic or Oriental)
*Pristaulacus memnonius* Sun & Sheng, 2007	Henan
*Pristaulacus nobilei* Turrisi & Smith, 2011	Jiangsu, Guangdong, Hongkong, Macao
*Pristaulacus pieli* Kieffer, 1924	Jiangxi
*Pristaulacus porcatus* Sun & Sheng, 2007	Henan
*Pristaulacus rufipes* Enderlein, 1912	Taiwan
*Pristaulacus zhejiangensis* He & Ma, 2002	Zhejiang

The extensive search for aulacid-specimens in several museums of China as well as relevant material from European museums resulted in the discovery of a total of 25 species, 6 of which are newly described, one *Aulacus* and five *Pristaulacus*. The present paper is the first attempt to revise the Chinese Aulacidae as a framework for further possible contributions.

## Material and methods

Descriptions of the species have been made under either an Olympus SZ61 or SZ40 stereomicroscope, with lighting achieved through a 40W LED lamp or a 27W fluorescent lamp. Photographic images were produced by a digital microscope (VHX-2000c, KEYENCE, Osaka, Japan), and plates were finished with ACDSee 10.0 and Photoshop CS 8.0.1, mostly to adjust the size and background.

Morphological nomenclature follows Crosskey (1951), Huber and Sharkey (1993), and Gauld and Bolton (1996). Terminology for surface sculpture follows Harris (1979). For the number of tooth-like processes on inner margin of pretarsal claw, apex is not included since it represents the tip of the claw (Turrisi 2007).

In text, the following abbreviations are used for some morphological structures: A = antennomere; OOL = distance between outer margin of posterior ocellus and eye; POL = distance between inner margins of posterior ocelli; T = Tergite; S = Sternite.

Type material and other specimens have been examined from the following institutions:

BMNH
 The Natural History Museum, London, UK (Ms. Suzanne Ryder) 


BPBM
 Bernice P. Bishop Museum, Honolulu (Hawaii), U.S.A. (Dr. Francis G. Howarth) 


CAS
California Academy of Sciences, San Francisco, California, U.S.A. (Dr. Wojciech J. Pulawski) 


HNHM
Hungarian Natural History Museum, Budapest, Hungary (Dr. Csosz Sandor) 


IZCAS
Institute of Zoology, Chinese Academy of Sciences, Beijing, China (Dr. Jun Chen, Mr. Jian Yao, Mrs. Hong Liu) 


LACM
 Los Angeles County Museum of Natural History, Los Angeles, California, U.S.A. (through courtesy of Dr. David R. Smith) 


OLML
 Oberosterreichisches Landesmuseum, Linz, Austria (Dr. Fritz Gusenleitner) 


NHRS
Swedish Museum of Natural History, Department of Entomology, Stockholm, Sweden (Dr. Hege Vårdal) 


SCAU
 Hymenopteran Collection, South China Agricultural University, Guangzhou, China (Dr. Zai-fu Xu) 


SDEI
Deutsches Entomologisches Institut, Müncheberg, Germany (Prof. Joachim Oehlke, Dr. Andreas Taeger) 


SEMC
 Shanghai Entomological Museum, Shanghai, China (Dr. Hai-sheng Ying) 


SFPS
 General Station of Forest Pest Management, State Forestry Administration, Shenyang, China (Prof. Mao-Ling Sheng) 


TCUC
 Turrisi G.F. Collection, University of Catania, Catania, Italy 


USNM
National Museum of Natural History, Smithsonian Institution, Washington, DC, USA (Dr. David R. Smith) 


ZJU
 Department of Plant Protection, Zhejiang University, Hangzhou, China (Prof. Jun-hua He & Prof. Xue-xin Chen) 


ZMHB
Museum für Naturkunde der Humboldt-Universitӓt, Berlin, Germany (Dr. Frank Koch) 


## Systematics

### Key to Chinese genera of Aulacidae

**Table:** 

1	Occipital carina absent (Fig. 5); forewing with cross-vein 2r-m, with 2-rs+m long (as in Fig. 8), almost as long as 1sr+m; hind coxa of female without groove or notch on inner lateral surface (Fig. 9); pretarsal claw not pectinate, without tooth-like processes along inner margin (Fig. 11)	***Aulacus* Jurine**
–	Occipital carina present (Figs 16, 17, 27, 28, 39, 40, 49, 60, 71, 79, 80, 92, 103, 104, 114, 115); forewing without cross-vein 2r-m, with 2-rs+m relatively short (as in Figs 95, 107, 118) or extremely short (as in Figs 20, 31, 43, 74, 83); hind coxa of female with groove or notch on inner lateral surface (Figs 32, 44, 96, 108); pretarsal claw pectinate with two to six distinct tooth-like processes along inner margin (Figs 22, 37, 54, 64, 68, 85, 97, 101, 120)	***Pristaulacus* Kieffer**

**Figure 1.**
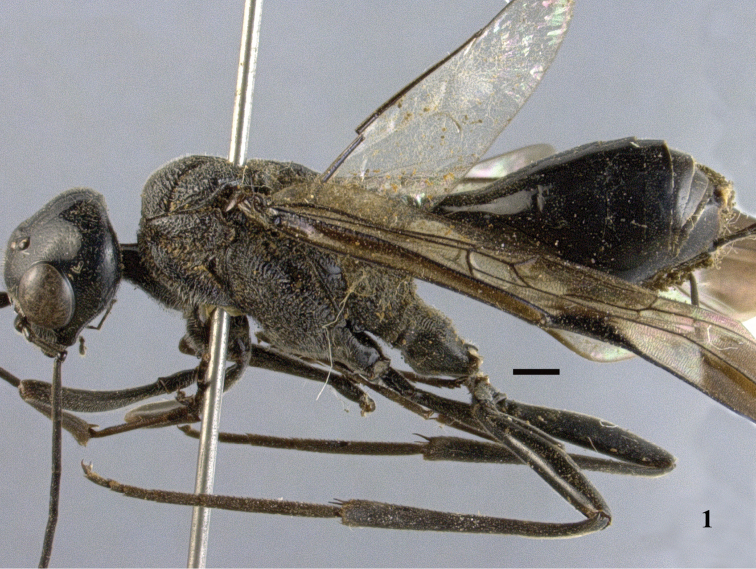
*Aulacus magnus* sp. n., holotype, female, habitus, lateral. Scale bar: 1 mm.

**Figures 2–7.**
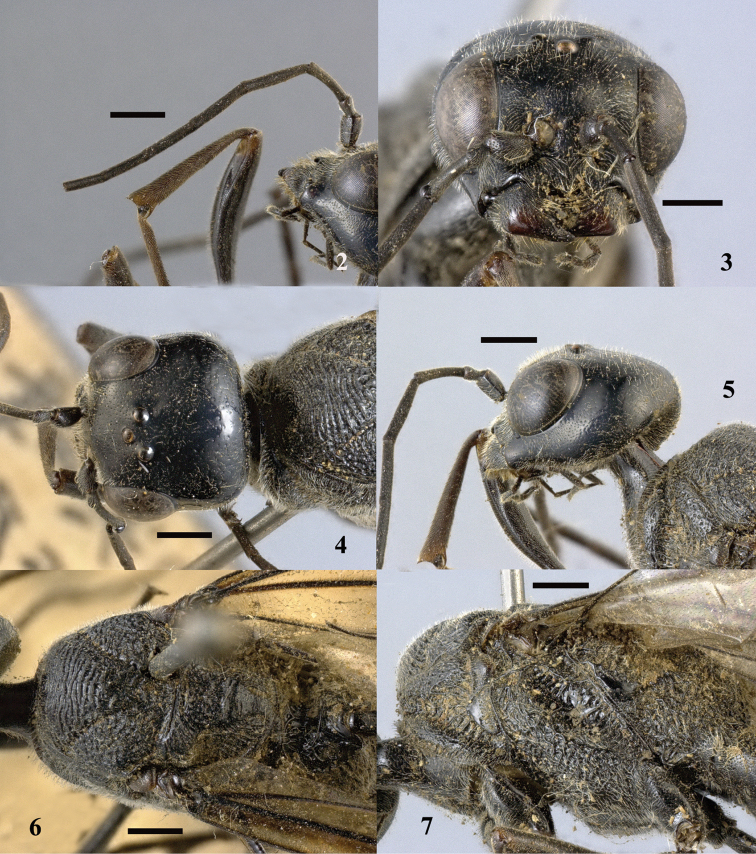
*Aulacus magnus* sp. n., holotype, female. **2** Antenna **3** head anterior **4** head dorsal **5** head lateral **6** mesosoma dorsal **7** mesosoma lateral. Scale bar: 1 mm.

**Figures 8–12.**
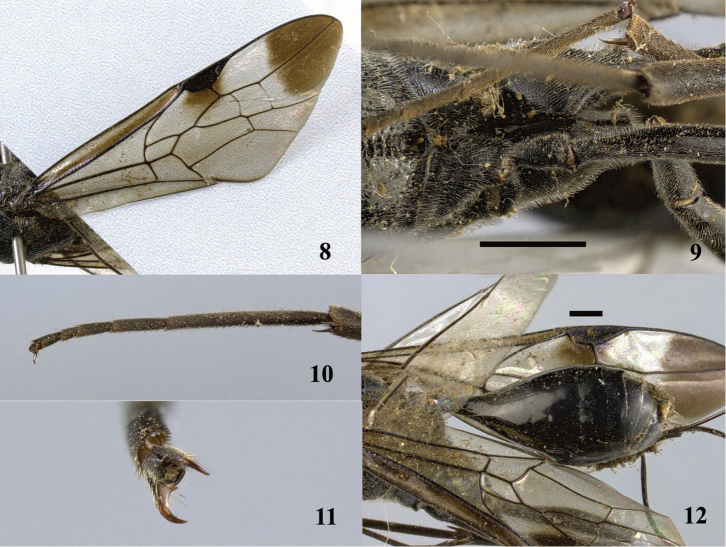
*Aulacus magnus* sp. n., holotype, female. **8** Forewing **9** hind coxae **10** hind tarsus **11** pretarsal claws **12** metasoma dorso-lateral. Scale bar: 1 mm.

**Figure 13.**
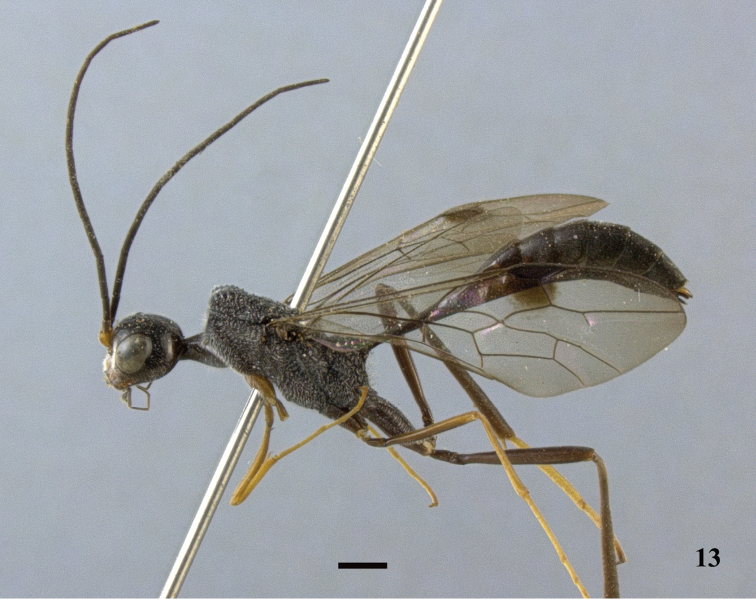
*Pristaulacus calidus* sp. n., holotype, male, habitus, lateral. Scale bar: 1 mm.

**Figures 14–19.**
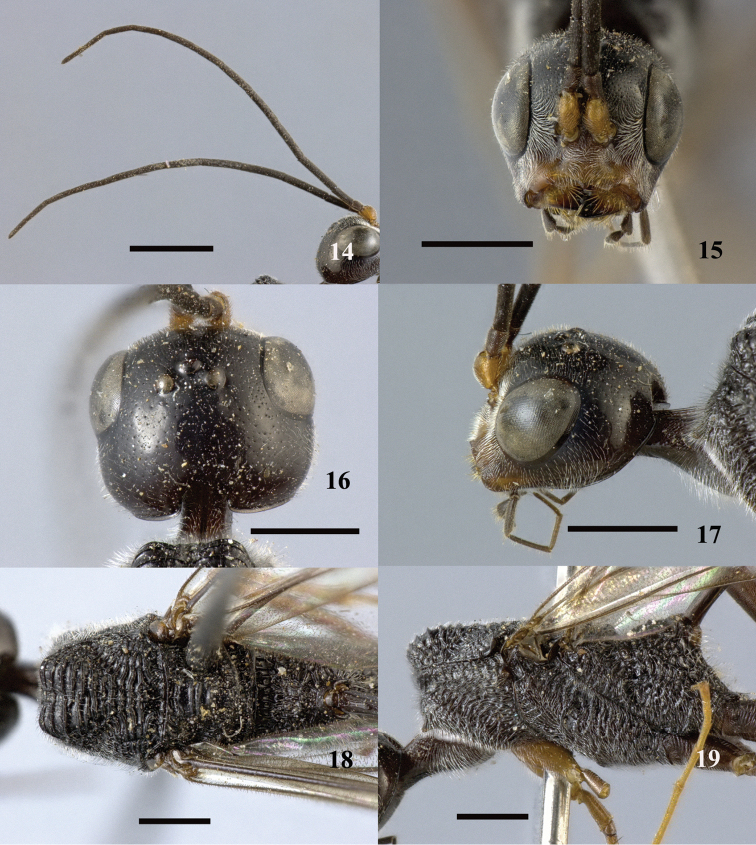
*Pristaulacus calidus* sp. n., holotype, male. **14** Antennae **15** head anterior **16** head dorsal **17** head lateral **18** mesosoma dorsal **19** mesosoma lateral. Scale bar: 1 mm.

**Figures 20–23.**
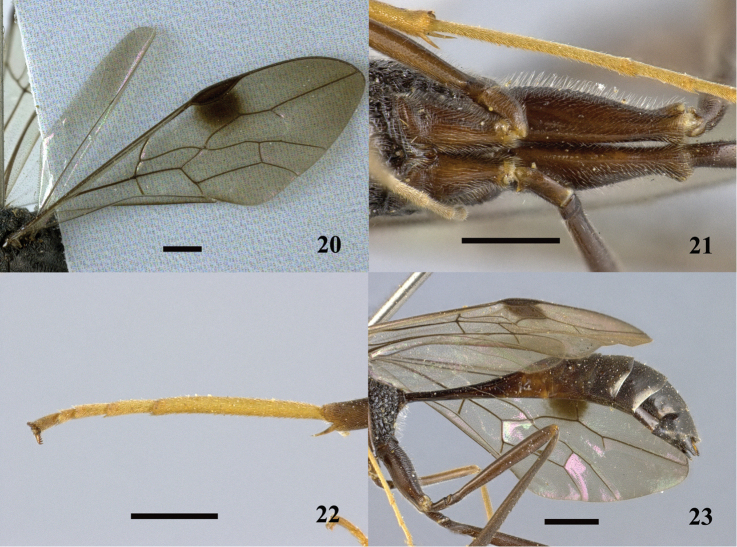
*Pristaulacus calidus* sp. n., holotype, male. **20** Forewing **21** hind coxae **22** hind tarsus **23** metasoma lateral. Scale bar: 1 mm.

**Figure 24.**
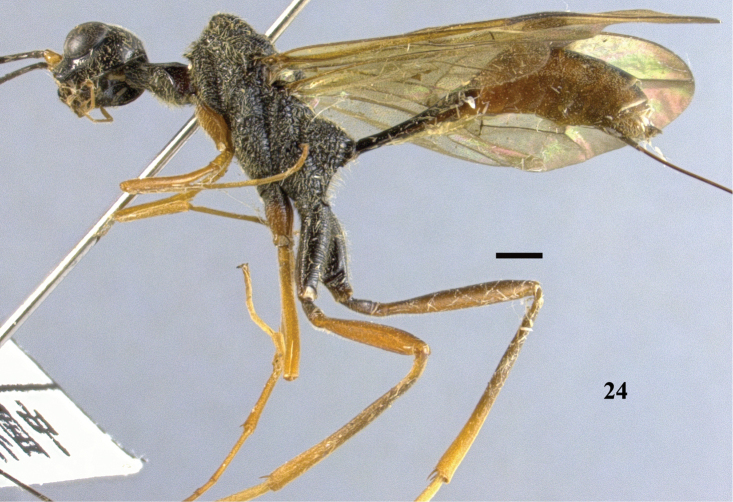
*Pristaulacus centralis* sp. n., holotype, female, habitus, lateral. Scale bar: 1 mm.

**Figures 25–30.**
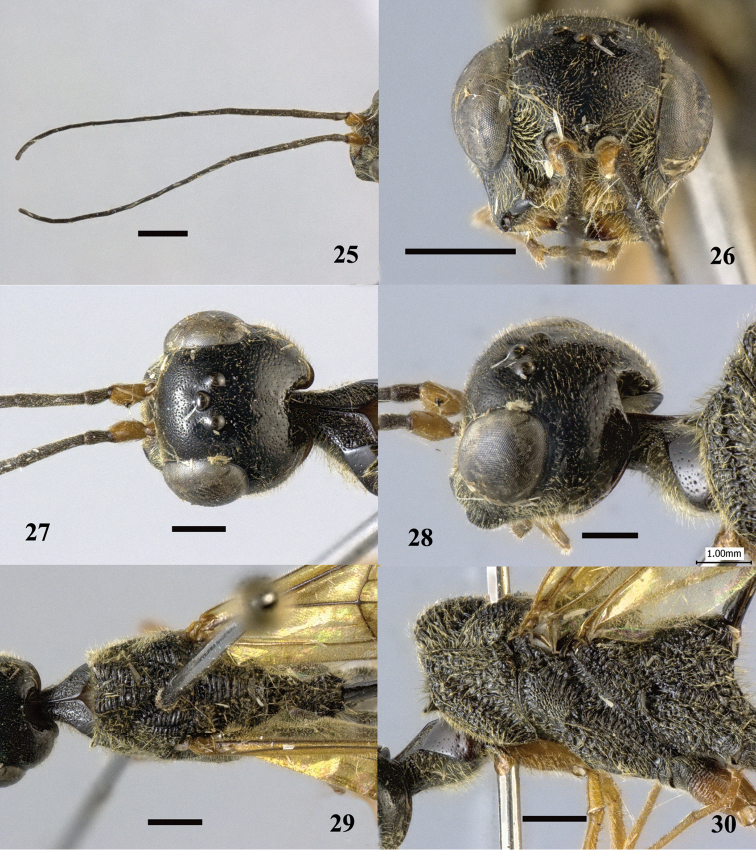
*Pristaulacus centralis* sp. n., holotype, female. **25** Antennae **26** head anterior **27** head dorsal **28** head lateral **29** mesosoma dorsal **30** mesosoma lateral. Scale bar: 1 mm.

**Figures 31–34.**
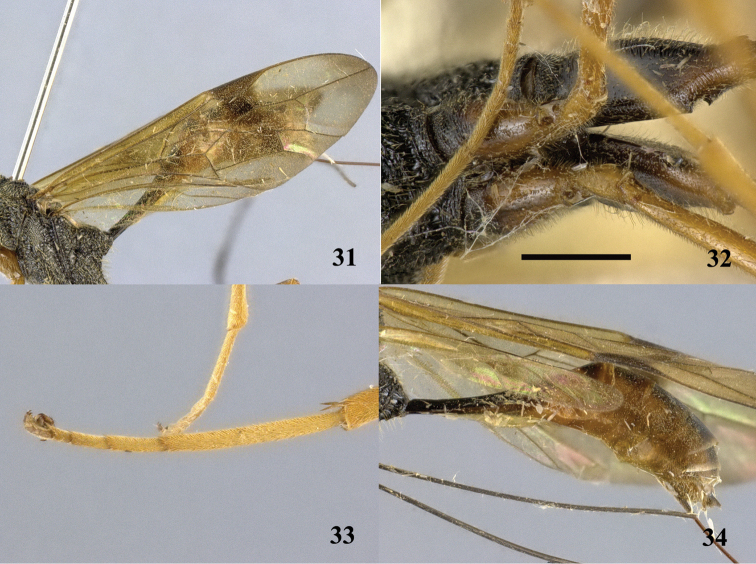
*Pristaulacus centralis* sp. n., holotype, female. **31** Forewing **32** hind coxae **33** hind tarsus **34** metasoma lateral. Scale bar: 1 mm.

**Figure 35.**
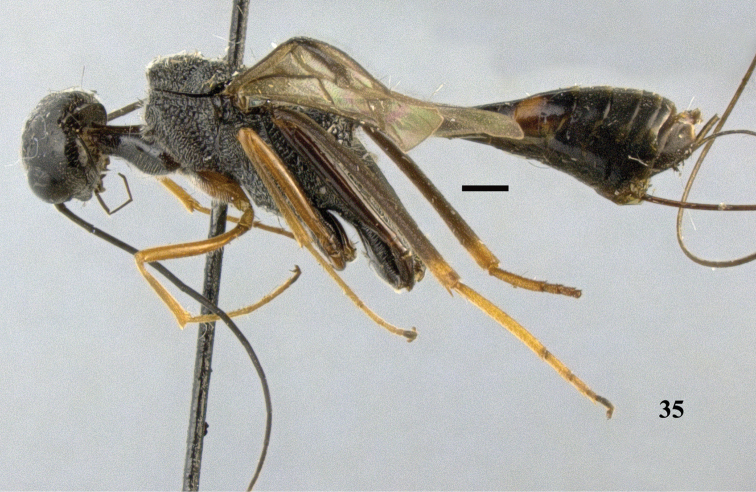
*Pristaulacus comptipennis* Enderlein, 1912, female, habitus, lateral. Scale bar: 1 mm.

**Figures 36–42.**
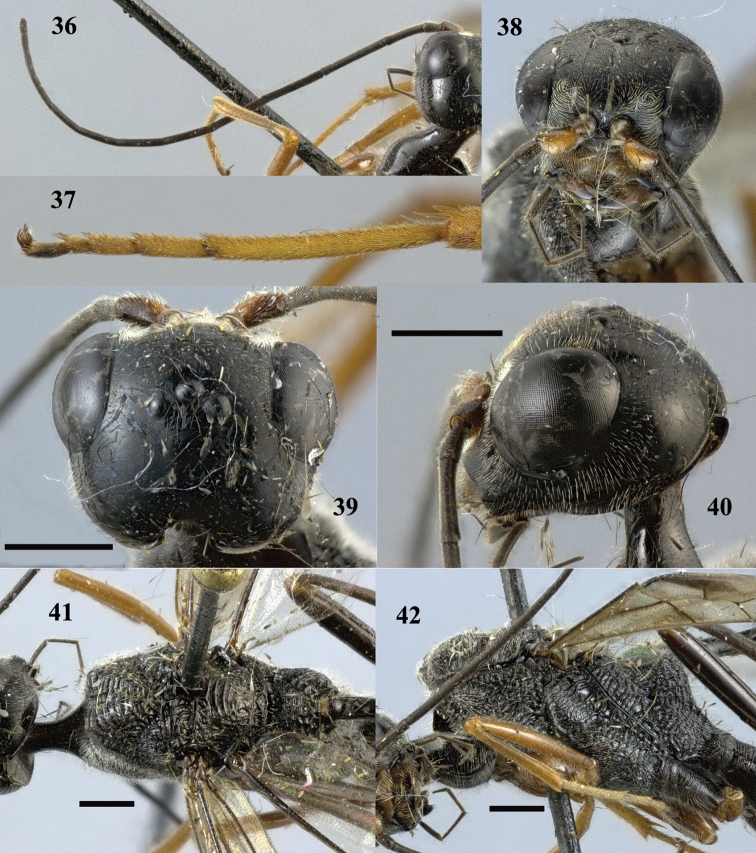
*Pristaulacus comptipennis* Enderlein, 1912, female. **36** Antenna **37** hind tarsus **38** head anterior **39** head dorsal **40** head lateral **41** mesosoma dorsal **42** mesosoma lateral. Scale bar: 1 mm.

**Figures 43–44.**
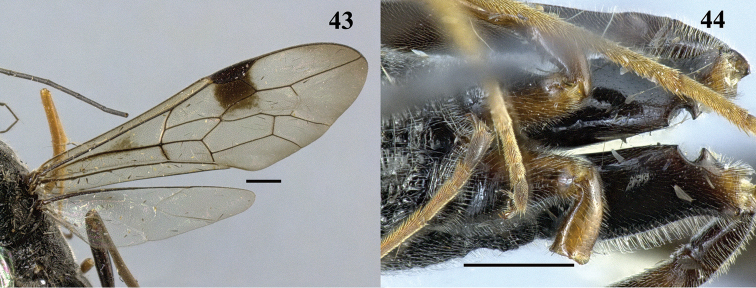
*Pristaulacus comptipennis* Enderlein, 1912, female. **43** Forewing and hind wing **44** hind coxae. Scale bar: 1 mm.

**Figure 45.**
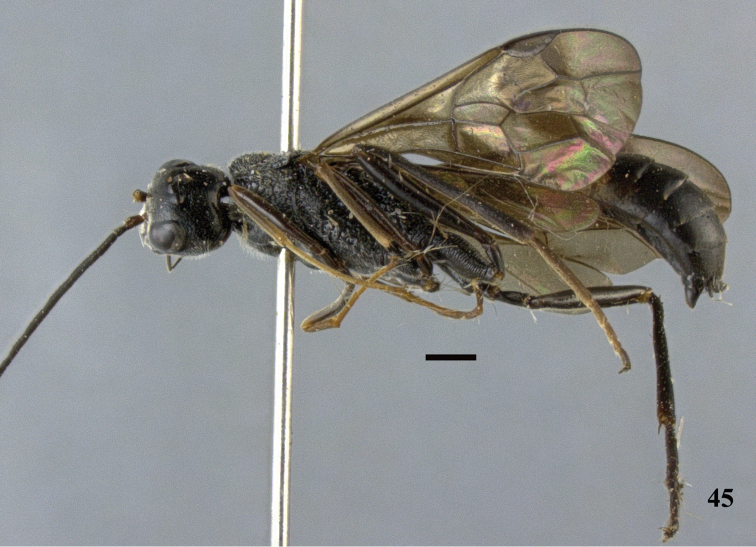
*Pristaulacus excisus* Turner, 1922, male, habitus, lateral. Scale bar: 1 mm.

**Figures 46–51.**
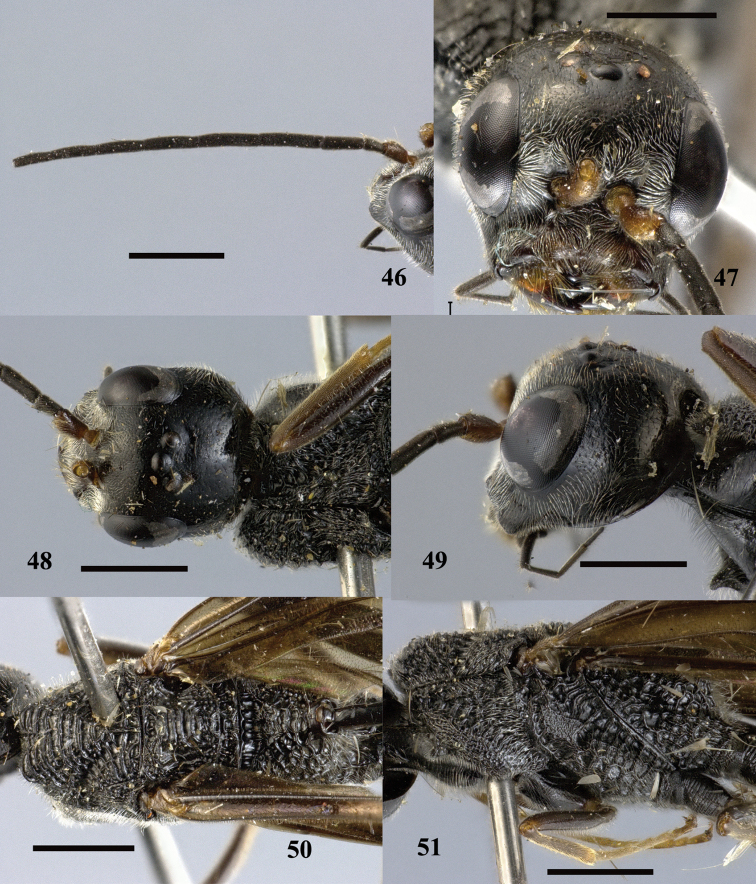
*Pristaulacus excisus* Turner, 1922, male. **46** Antenna **47** head anterior **48** head dorsal **49** head lateral **50** mesosoma dorsal **51** mesosoma lateral. Scale bar: 1 mm.

**Figures 52–55.**
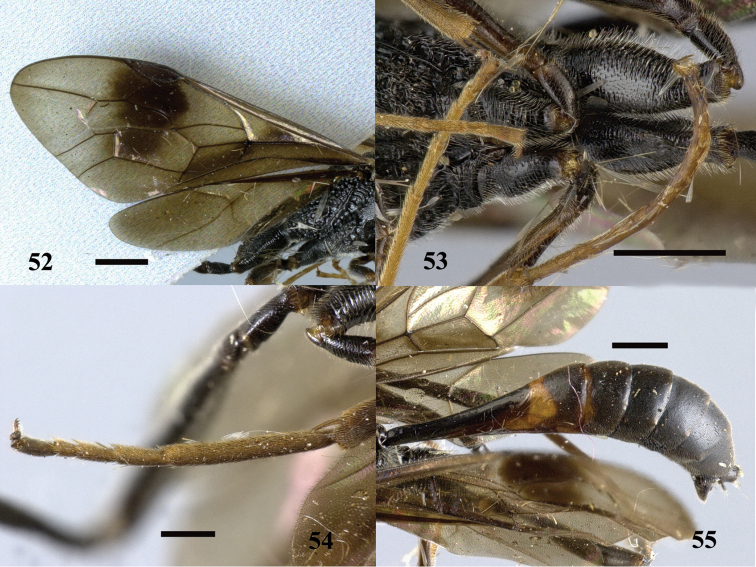
*Pristaulacus excisus* Turner, 1922, male. **52** Forewing and hind wing **53** hind coxae **54** hind tarsus **55** metasoma lateral. Scale bar: 1 mm.

**Figure 56.**
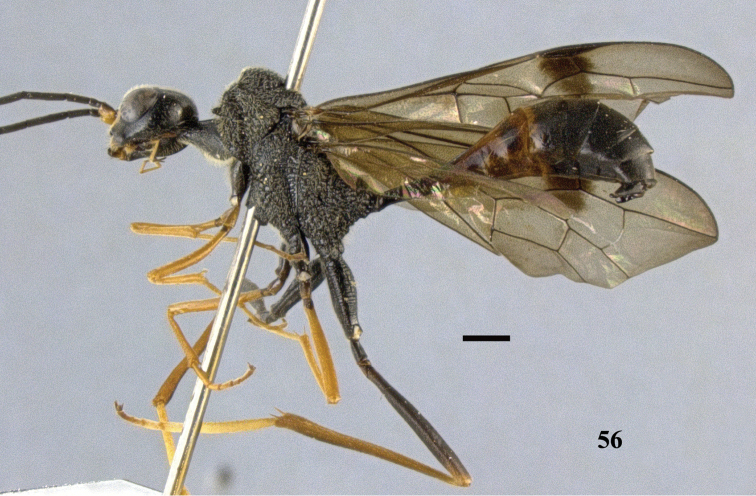
*Pristaulacus fopingi* sp. n., holotype, male, habitus, lateral. Scale bar: 1 mm.

**Figures 57–62.**
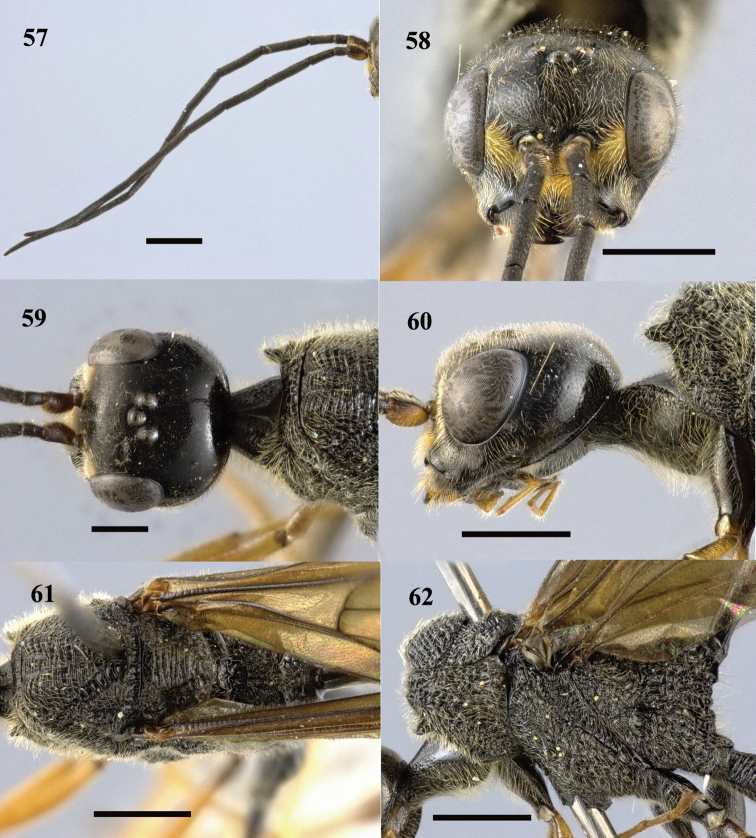
*Pristaulacus fopingi* sp. n., holotype, male. **57** Antennae **58** head anterior **59** head dorsal **60** head lateral **61** mesosoma dorsal **62** mesosoma lateral. Scale bar: 1 mm.

**Figures 63–65.**
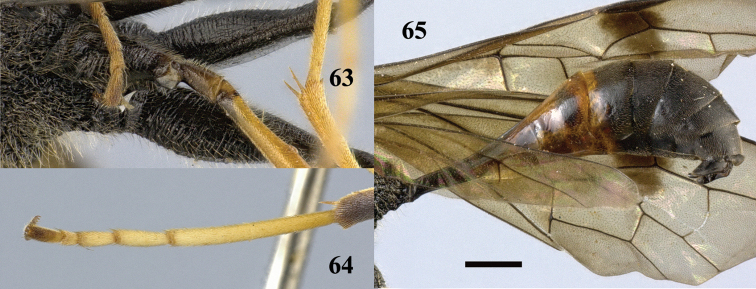
*Pristaulacus fopingi* sp. n., holotype, male. **63** Hind coxae **64** hind tarsus **65** metasoma lateral. Scale bar: 1 mm.

**Figure 66.**
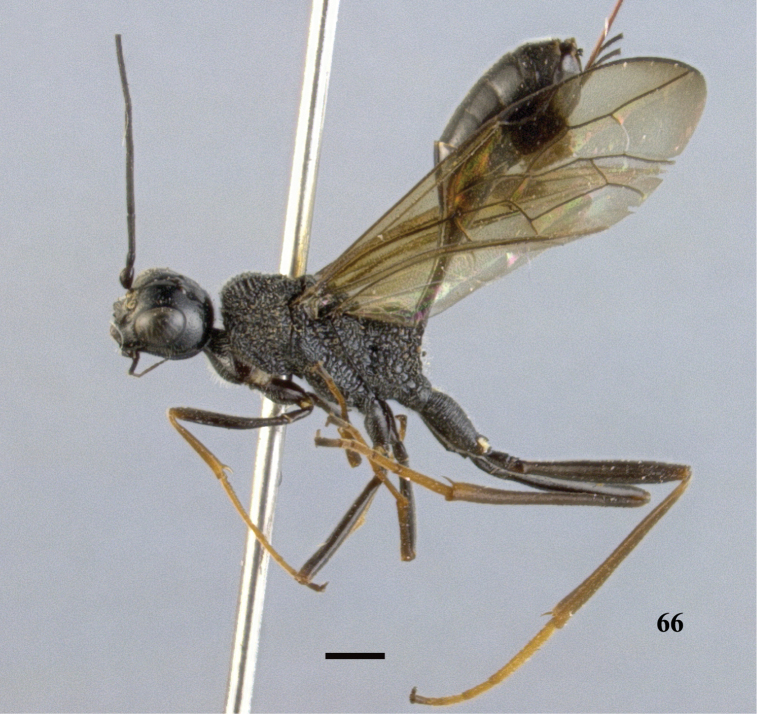
*Pristaulacus intermedius* Uchida, 1932, female, habitus, lateral. Scale bar: 1 mm.

**Figures 67–73.**
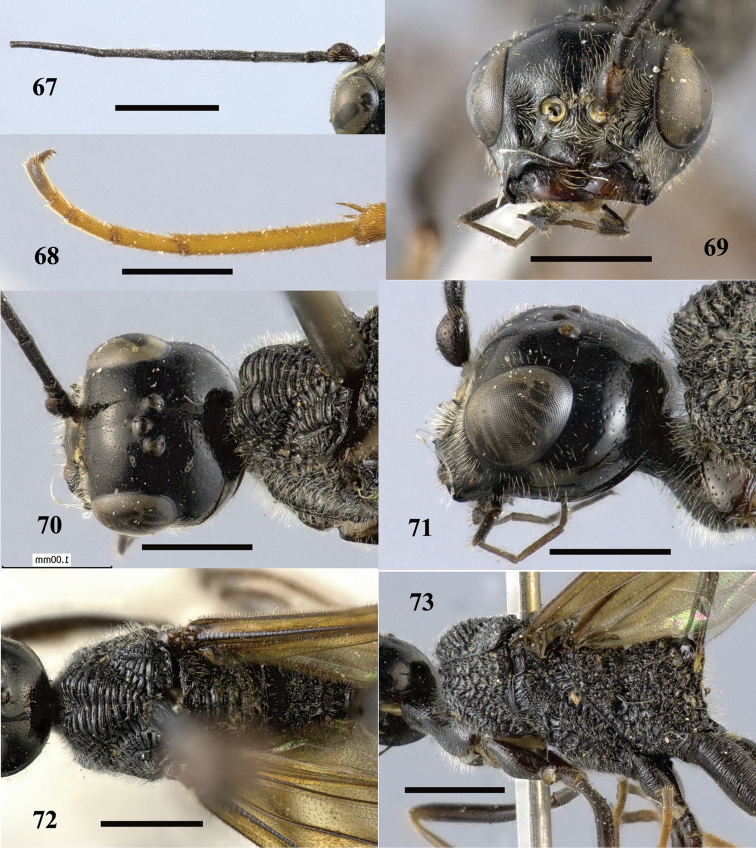
*Pristaulacus intermedius* Uchida, 1932, female. **67** Antenna **68** hind tarsus **69** head anterior **70** head dorsal **71** head lateral **72** mesosoma dorsal **73** mesosoma lateral. Scale bar: 1 mm.

**Figures 74–75.**
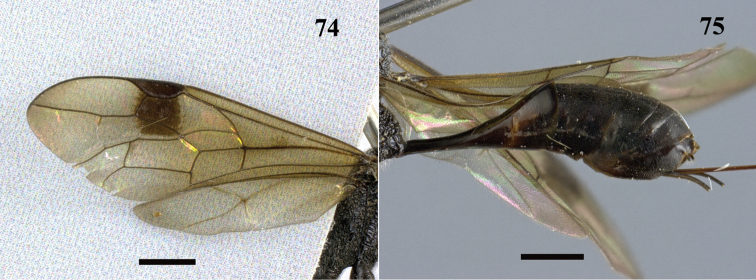
*Pristaulacus intermedius* Uchida, 1932, female. **74** Forewing and hind wing **75** metasoma lateral. Scale bar: 1 mm.

**Figure 76.**
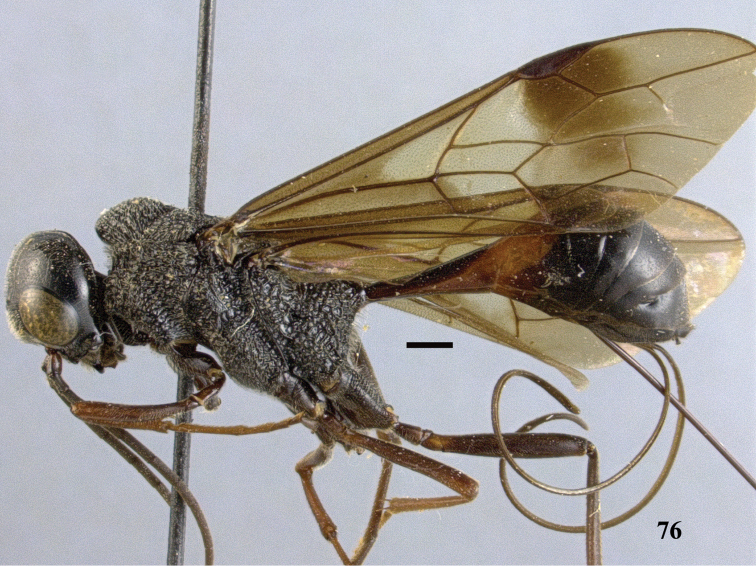
*Pristaulacus iosephi* Turrisi & Madl, 2013, female, habitus, lateral. Scale bar: 1 mm.

**Figures 77–82.**
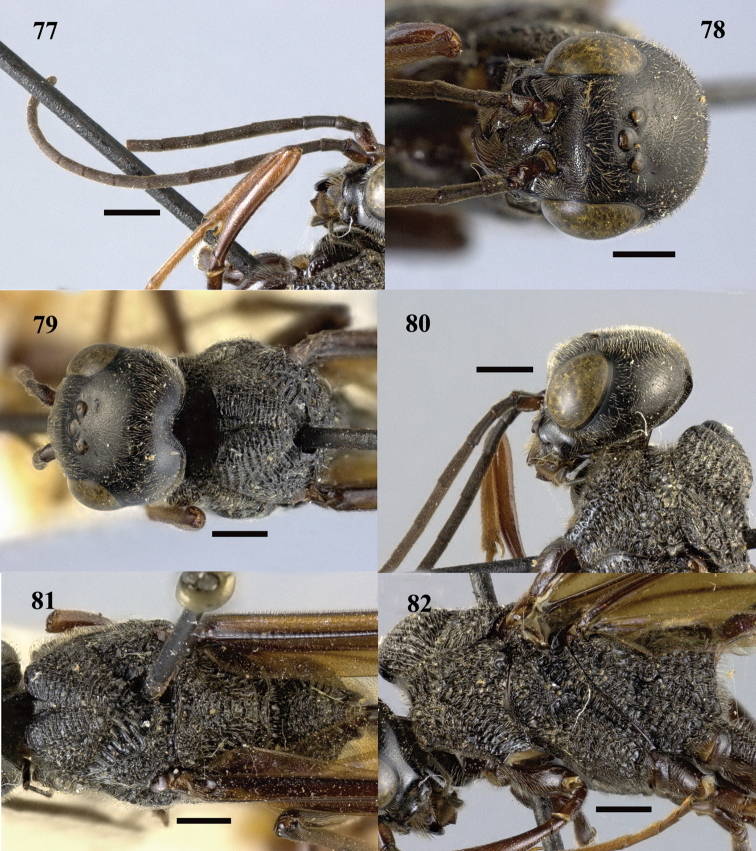
*Pristaulacus iosephi* Turrisi & Madl, 2013, female. **77** Antennae **78** head anterior **79** head dorsal **80** head lateral **81** mesosoma dorsal **82** mesosoma lateral. Scale bar: 1 mm.

**Figures 83–87.**
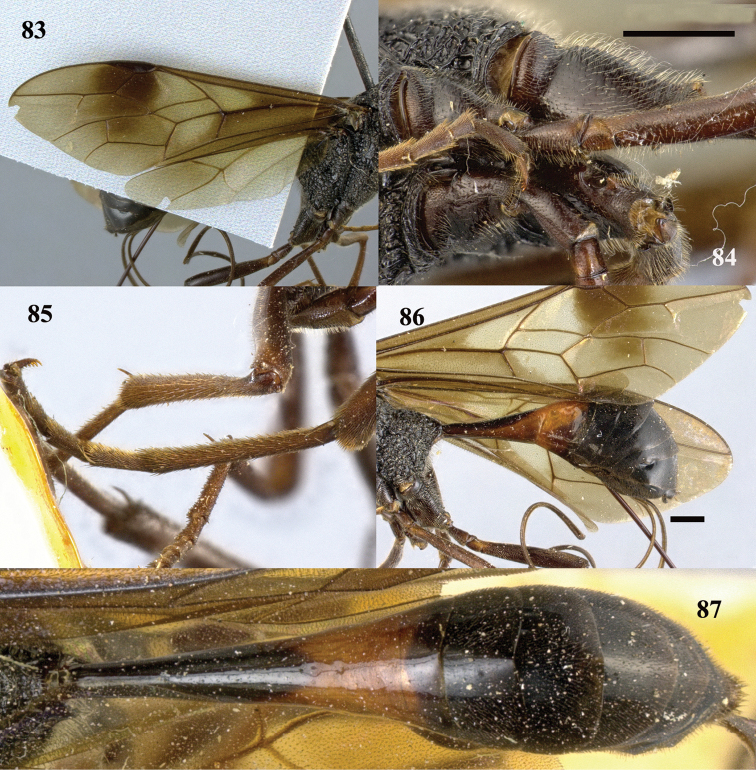
*Pristaulacus iosephi* Turrisi & Madl, 2013, female. **83** Forewing and hind wing **84** hind coxae **85** hind tarsus **86** metasoma lateral **87** metasoma dorsal. Scale bar: 1 mm.

**Figure 88.**
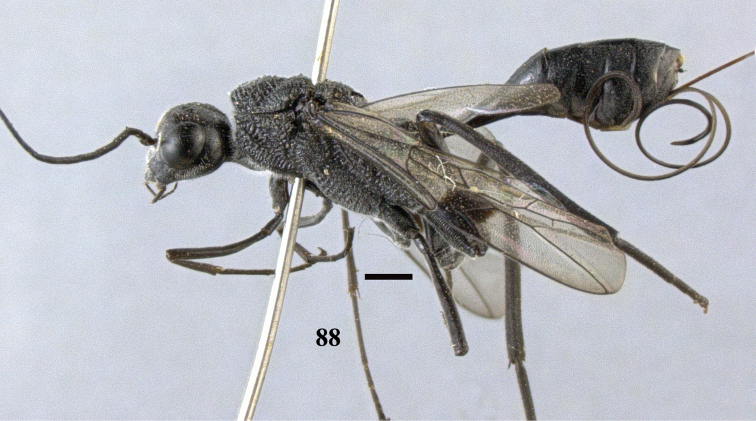
*Pristaulacus obscurus* sp. n., holotype, female, habitus, lateral. Scale bar: 1 mm.

**Figures 89–94.**
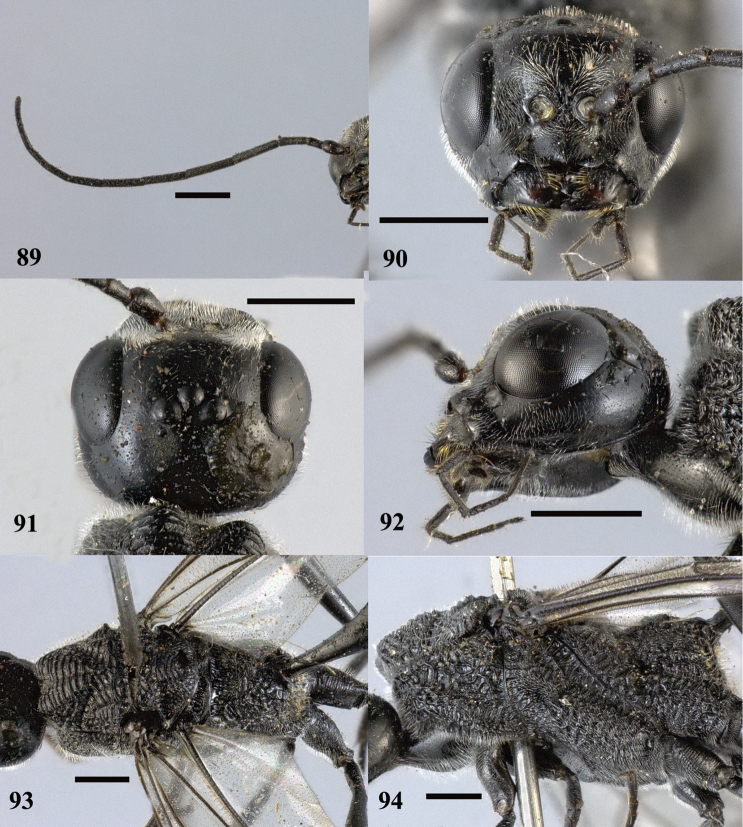
*Pristaulacus obscurus* sp. n., holotype, female. **89** Antenna **90** head anterior **91** head dorsal **92** head lateral **93** mesosoma dorsal **94** mesosoma lateral. Scale bar: 1 mm.

**Figures 95–98.**
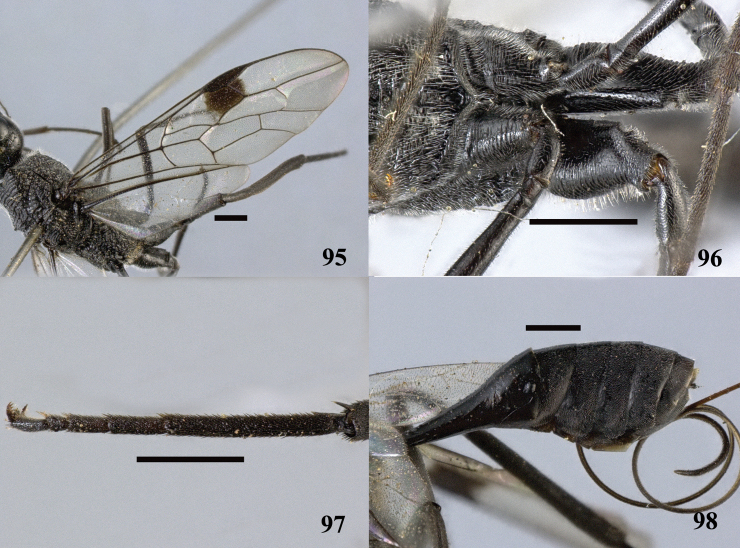
*Pristaulacus obscurus* sp. n., holotype, female. **95** Forewing **96** hind coxae **97** hind tarsus **98** metasoma lateral. Scale bar: 1 mm.

**Figure 99.**
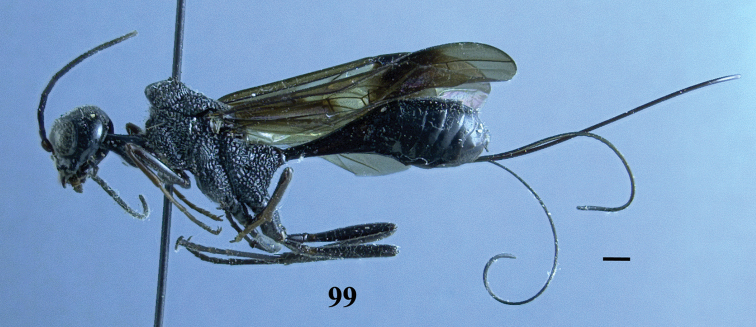
*Pristaulacus pseudoiosephi* sp. n., paratype, female, habitus, lateral. Scale bar: 1 mm.

**Figures 100–106.**
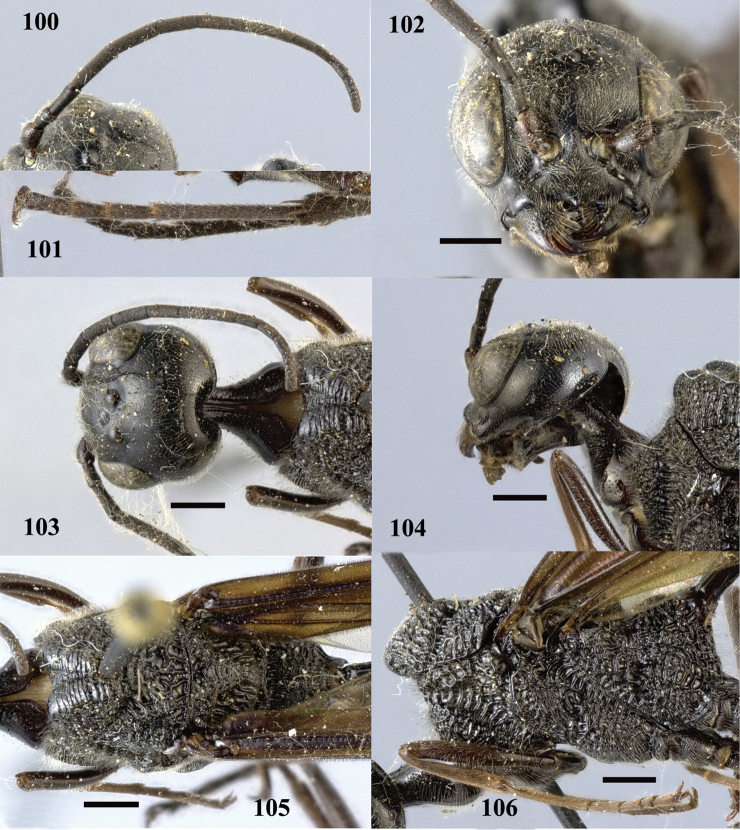
*Pristaulacus pseudoiosephi* sp. n., paratype, female. **100** Antenna **101** hind tarsi **102** head anterior **103** head dorsal **104** head lateral **105** mesosoma dorsal **106** mesosoma lateral. Scale bar: 1 mm.

**Figures 107–110.**
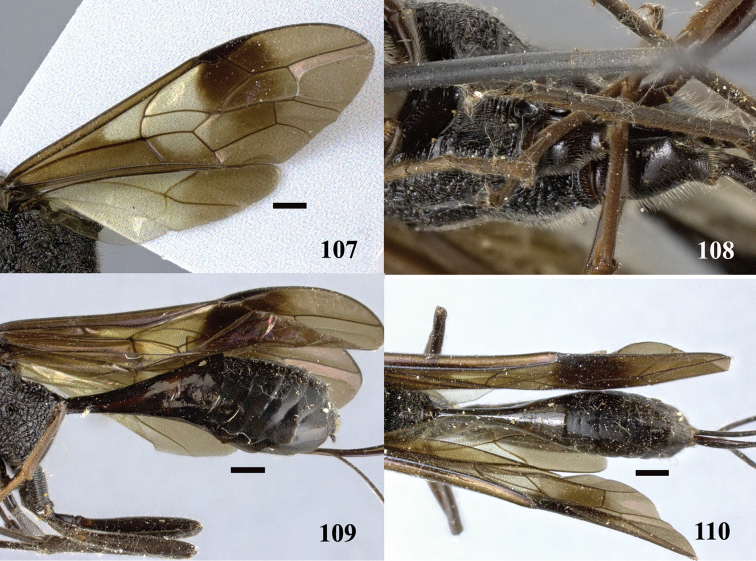
*Pristaulacus pseudoiosephi* sp. n., paratype, female. **107** Forewing and hind wing **108** hind coxae **109** metasoma lateral **110** metasoma dorsal. Scale bar: 1 mm.

**Figure 111.**
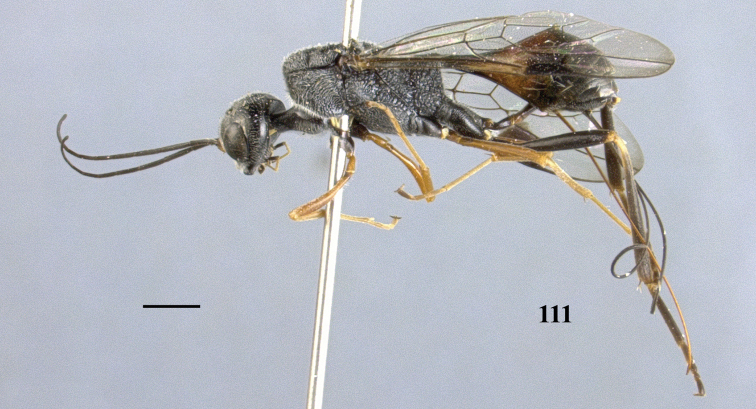
*Pristaulacus rufobalteatus* Cameron, 1907, female, habitus, lateral. Scale bar: 1 mm.

**Figures 112–117.**
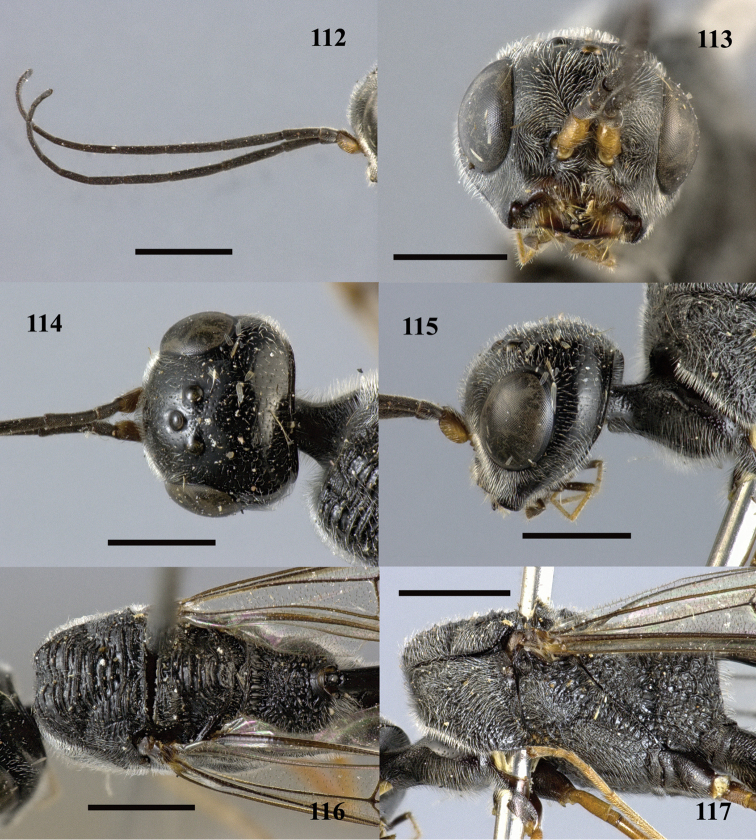
*Pristaulacus rufobalteatus* Cameron, 1907, female. **112** Antennae **113** head anterior **114** head dorsal **115** head lateral **116** mesosoma dorsal **117** mesosoma lateral. Scale bar: 1 mm.

**Figures 118–121.**
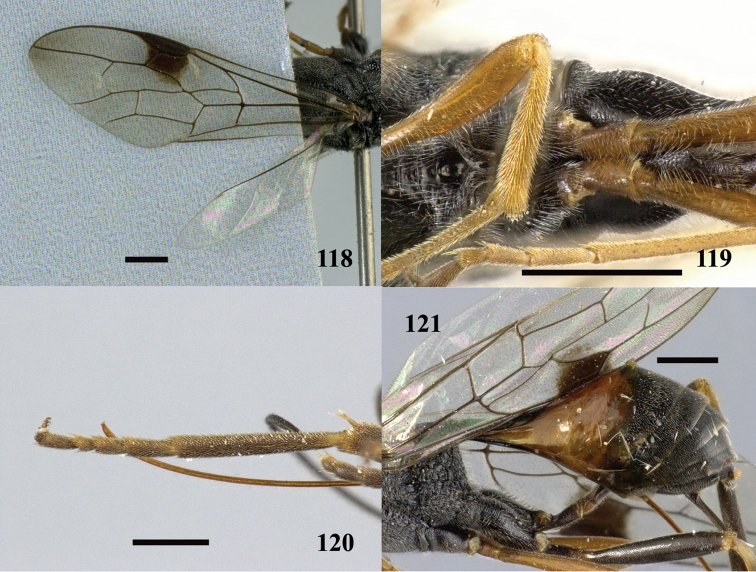
*Pristaulacus rufobalteatus* Cameron, 1907, female. **118** Forewing and hind wing **119** hind coxae **120** hind tarsus **121** metasoma lateral. Scale bar: 1 mm.

#### Genus Aulacus

Taxon classificationAnimaliaHymenopteraAulacidae

Jurine, 1807

Aulacus Jurine, 1807: 89. Type species: Aulacus striatus Jurine, by monotypy.
Aulacus Jurine: Blanchard 1840: 300; Bradley 1908: 120; Kieffer 1912: 344; Hedicke 1939: 4; Konishi 1990: 641; Alekseev 1995: 39; Smith 2001: 277; Turrisi et al. 2009.

##### Remarks.

The genus *Aulacus* has been demonstrated to be paraphyletic (Turrisi et al. 2009), and to date lacks a comprehensive revision of the taxa included to ascertain phylogenetic relationships.

##### Key to Chinese species of *Aulacus*

**Table:** 

1	Metasoma entirely black	**2**
–	Metasoma at least with 2^nd^ and 3^rd^ tergites brown or reddish-brown	**3**
2	Antenna black (Fig. 2); forewing with large dark brown spot under stigma and at apex (Fig. 8)	***Aulacus magnus* sp. n.**
–	Antenna extensively reddish-orange, with A1–A4 and A11–A14 dark orange; forewing without dark brown spots	***Aulacus schoenitzeri* Turrisi**
3	Head mainly black with malar area and gena brown	***Aulacus striatus* Jurine**
–	Head mainly reddish-brown, with upper part of frons and median part of vertex black	**4**
4	Fore coxa brown; lower part of frons with sparse punctures, upper part with oblique transverse carinulae	***Aulacus flavigenis* Alekseev**
–	Fore coxa black; lower part of frons transverse-carinate, upper part punctate	***Aulacus sinensis* He & Chen**

#### Aulacus flavigenis

Taxon classificationAnimaliaHymenopteraAulacidae

Alekseev, 1986

Fig. 122

Aulacus flavigenis Alekseev, 1986: 17.
Aulacus salicius Sun & Sheng, 2007b: 122. Synonymized by Sundukov and Lelej (2015).
Aulacus salicius Sun & Sheng: Turrisi et al. 2009: 56; Smith and Tripotin 2011: 520.

##### Material examined.

No available material from China for this study. Examined material: 1 ♀ from South Korea (Tripotin P., gift to Turrisi G.F.).

##### Diagnosis.

Head mainly reddish-brown, with upper part of frons and median part of vertex black; fore coxa brown; metasoma black with most of first tergite (except base) and second tergite reddish-brown; lower part of frons with sparse and indistinct punctures, upper part with distinct oblique transverse carinulae; ovipositor about 0.8 × forewing length.

##### Distribution.

China (Heilongjiang); Russia (Primorski Krai and Skotovo) (Alekseev 1986; Sundukov and Lelej 2015); South Korea (Gangwon-do) (Smith and Tripotin 2011).

##### Biology.

Collected in June (Sun and Sheng 2007b). Host: *Xiphydria palaeanarctica* Semenov-Tian-Shanskij (Hymenoptera, Xiphydriidae) (Smith and Tripotin 2011), *Xiphydria popovi* Semenov-Tian-Shanskij & Gussakovskij (Sun and Sheng 2007b). Additional data on biology are provided by Smith and Tripotin (2011).

##### Remarks.

Redescription is provided by Sundukov and Lelej (2015).

#### Aulacus magnus sp. n.

Taxon classificationAnimaliaHymenopteraAulacidae

http://zoobank.org/3EB00C7D-A9F2-4DB8-A543-95AD3AAB4479

Figs 1 , 2–7 , 8–12 , 122

##### Material examined.

Holotype, ♀ (IZCAS), CHINA: Hainan, Mt. Jianfengling, 670 m, 6.V.1964, IOZ(E)1903950.

##### Etymology.

From the Latin adjective “*magnus*”, meaning “large”, a noun in apposition.

##### Diagnosis.

Antenna entirely black; forewing with large dark brown spots under stigma and at apex; head largely smooth with sparse and fine punctures; lateroventral margin of pronotum without teeth; scutellum mostly rugose with nearly smooth area posteriorly; pretarsal claw with one basal large tooth-like process; ovipositor 0.9 × forewing length.

##### Description.

Holotype. *Female*. Body length 16.2 mm; forewing length 14.0 mm.

*Colour*. Black except: apical half of mandible reddish-brown; forewing hyaline, with large dark brown spot under stigma and large dark brown spot at apex; hind wing hyaline.

*Head*. From above, 1.2 × wider than long, shiny; lower interocular distance 1.5 × eye height; malar space 0.3 × eye height; occipital margin straight; temple, from above, rounded, slightly longer than eye length; occipital carina 0.1 × diameter of an ocellus; POL:OOL=0.8; head largely smooth with sparse and fine punctures (distance between punctures 1.0–4.0 × diameter of a puncture); A3 5.0 × longer than wide; A4 6.0 × longer than wide, and 1.4 × longer than A3; A5 5.5 × longer than wide, and 1.3 × longer than A3.

*Mesosoma*. Pronotum largely rugose, coarsely areolate-rugose in middle, with lateroventral margin regularly rounded and without teeth; propleuron shiny and largely smooth with sparse and fine punctures; mesoscutum transverse-carinate anteriorly, areolate-rugose posterior to notaulus, prescutum not emarginate medially; notaulus shallow and narrow; scutellum mostly rugose with nearly smooth area posteriorly; axilla oblique-rugulose; metanotum irregularly rugose; propodeum coarsely areolate-rugose; mesopleuron and metapleuron coarsely areolate-rugose; forewing with vein 2-rs+m long: cells SM2 and D1 distantly separated; hind wing veins faint to absent; hind coxa with dorsal surface transverse-carinate basally, densely and finely punctate apically, and ventral surface rugulose-punctate to punctate, punctures coarse and dense; hind basitarsus 12.0 × longer than wide, 1.2 × longer than tarsomeres 2–5; pretarsal claw with one large basal tooth-like process.

*Metasoma*. Pyriform (lateral view), compressed laterally; petiole elongate, 7.0 × longer than wide; segments 1 and 2 polished and shiny; following segments with fine and dense punctures; ovipositor 0.9 × forewing length.

Male. Unknown.

##### Distribution.

China (Hainan).

**Figure 122.**
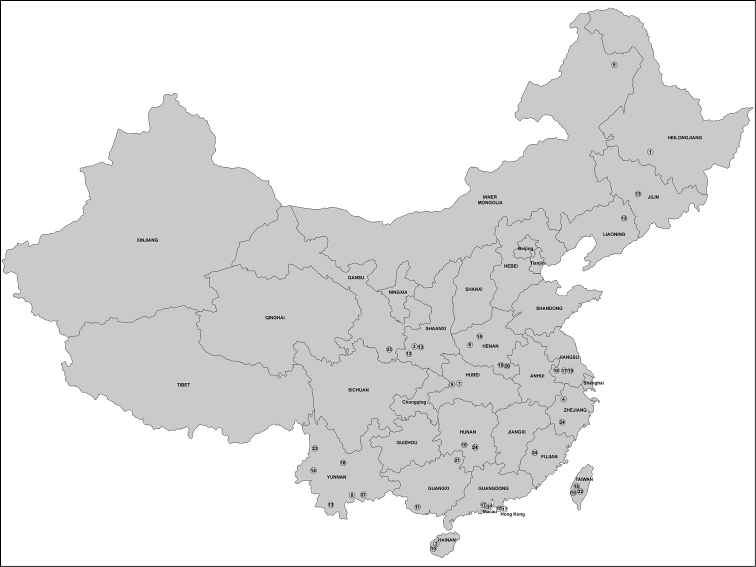
Distribution map of the species of Chinese Aulacidae (*Pristaulacus longicornis* Kieffer, 1911 is not included). **1** *Aulacus flavigenis* Alekseev, 1986 **2** *Aulacus magnus* sp. n. **3** *Aulacus schoenitzeri* Turrisi, 2005 **4** *Aulacus sinensis* He & Chen, 2007. **5** *Aulacus striatus* Jurine, 1807. **6** *Pristaulacus albitarsatus* Sun & Sheng, 2007 **7** *Pristaulacus asiaticus* Turrisi & Smith, 2011 **8** *Pristaulacus calidus* sp. n. **9** *Pristaulacus centralis* sp. n. **10** *Pristaulacus comptipennis* Enderlein, 1912. **11** *Pristaulacus excisus* Turner, 1922 **12** *Pristaulacus fopingi* sp. n. **13** *Pristaulacus intermedius* Uchida, 1932 **14** *Pristaulacus iosephi* Turrisi & Madl, 2013 **15** *Pristaulacus karinulus* Smith, 2001 **16** *Pristaulacus memnonius* Sun & Sheng, 2007 **17** *Pristaulacus nobilei* Turrisi & Smith, 2011 **18** *Pristaulacus obscurus* sp. n. **19** *Pristaulacus pieli* Kieffer, 1924 **20** *Pristaulacus porcatus* Sun & Sheng, 2007 **21** *Pristaulacus pseudoiosephi* sp. n. **22** *Pristaulacus rufipes* Enderlein, 1912 **23** *Pristaulacus rufobalteatus* Cameron, 1907 **24** *Pristaulacus zhejiangensis* He & Ma, 2002. Note: The South China Sea islands are not shown on this map.

##### Biology.

Collected in May. Host not known.

#### Aulacus schoenitzeri

Taxon classificationAnimaliaHymenopteraAulacidae

Turrisi, 2005

Fig. 122

Aulacus schoenitzeri Turrisi, 2005: 798.
Aulacus schoenitzeri Turrisi: Turrisi et al. 2009: 56.

##### Material examined.

Holotype, ♀ (OLML), CHINA: Shaanxi, Qinling, Xunyangba, 23.V–13.VI.1998, I. H. Marshal leg/*Aulacus schoenitzeri* Turrisi sp. n.

##### Diagnosis.

Antenna extensively reddish-orange with A1–A4 and A11–A14 darker; legs blackish, except tibiae and tarsi reddish-orange; metasoma entirely black; vertex dull, strongly striolate-punctate; ovipositor 0.9 × forewing length.

##### Distribution.

China (Shaanxi).

##### Biology.

Collected in May or June. Host not known.

#### Aulacus sinensis

Taxon classificationAnimaliaHymenopteraAulacidae

He & Chen, 2007

Fig. 122

Aulacus erythrogaster He & Chen, 2002: 149 (preoccupied by Aulacus erythrogaster Kieffer, 1904).
Aulacus sinensis He & Chen, 2007: 66 (replacement name for Aulacus erythrogaster He & Chen, 2002).
Aulacus sinensis He & Chen: Turrisi et al. 2009: 56; Turrisi 2013a: 332.

##### Material examined.

Holotype, ♀ (ZJU), CHINA: Zhejiang, Mt. Tianmu, 2–4.VI.1990, Xin-geng Wang, No. 903191/*Aulacus erythrogaster* He & Chen sp. n., 2002/ *Aulacus sinensis* He & Chen, nom. n., 2007.

##### Diagnosis.

Head mainly reddish brown, upper portion of frons and median portion of vertex black; fore and middle femora black, apically yellow, hind tibia yellow at basal 0.14, the rest blackish brown; frons punctate on upper half, transverse-striate on lower half; apical half of hind coxa with a longitudinal groove along inner side.

##### Distribution.

China (Zhejiang).

##### Biology.

Collected in June. Host not known.

#### Aulacus striatus

Taxon classificationAnimaliaHymenopteraAulacidae

Jurine, 1807

Fig. 122

Aulacus striatus Jurine, 1807: 89–90.
Aulacus striatus Jurine: Sun and Sheng 2007b: 124; Turrisi et al. 2009: 56; Broad and Livermore 2014: 2.

##### Material examined.

No available material from China for this study.

##### Diagnosis.

Antenna entirely blackish-brown; femora, tibiae and tarsi extensively reddish-orange; metasoma extensively reddish-orange; vertex shining, irregularly, coarsely and deeply punctured, sometimes with very fine carinulae; propodeum weakly declivous; ovipositor 0.7–0.8 × forewing length.

##### Distribution.

China (Inner Mongolia) (Sun and Sheng 2007b); Europe (Smith 2001; Broad and Livermore 2014).

##### Biology.

Collected in August (Sun and Sheng 2007b). Host not known.

##### Remarks.

The diagnosis is based on European specimens. Unfortunately, we were unable to examine Sun & Sheng’s specimens. Therefore, the status of this species in China is unclear to us.

#### Genus Pristaulacus

Taxon classificationAnimaliaHymenopteraAulacidae

Kieffer, 1900

Pristaulacus Kieffer, 1900: 813. Type species: Pristaulacus chlapowskii Kieffer, designated by Kieffer, 1903: 455.
Pristaulacus Kieffer: Kieffer 1903: 455; 1910: 350; 1911: 215; 1912: 376; Bradley 1908: 212; Hedicke 1939: 4; Koslov 1988: 243; Konishi 1990: 641; Alekseev 1995: 39; Smith 2001: 277; Turrisi 2006: 28; Turrisi 2007: 28; Turrisi et al. 2009: 53; Watanabe et al. 2013: 188.

##### Key to Chinese species of *Pristaulacus*

**Table:** 

1	Hind margin of head straight or weakly concave, without medial groove (Figs 59, 72, 91, 114); occipital carina not interrupted (Figs 59, 72, 91, 114)	**2**
–	Hind margin of head more or less grooved medially (Figs 16, 27, 39, 48, 79, 103); occipital carina interrupted (Figs 16, 27, 39, 48, 79, 103)	**12**
2	Lateroventral margin of pronotum without tooth-like process	***Pristaulacus pieli* Kieffer**
–	Lateroventral margin of pronotum at least with one tooth-like process (Figs 19, 30, 42, 51, 62, 73, 82, 94, 104, 117)	**3**
3	Hind basitarsus 1.9 × longer than tarsomeres 2–5	***Pristaulacus rufipes* Enderlein**
–	Hind basitarsus at most 1.3 × longer than tarsomeres 2–5	**4**
4	Occipital carina wide, 0.5 × diameter of ocellus, lamelliform, brownish	**5**
–	Occipital carina at most 0.2 × diameter of ocellus, pad-shaped, blackish	**7**
5	Large sized species (body length, excluding ovipositor about 15.0 mm); basal antennomeres very elongate (A3 8.3 × longer than wide, A4 14.0 × longer than wide)	***Pristaulacus longicornis* Kieffer**
-	Medium sized species (body length, excluding ovipositor about 10.0-11.0 mm); basal antennomeres elongate (A3 5.0–6.0 × longer than wide, A4 10.0 × longer than wide) (Fig. 67)	**6**
6	Metasoma entirely blackish, at most slightly lightened basally (Fig. 75)	***Pristaulacus intermedius* Uchida**
–	Metasoma extensively reddish orange	***Pristaulacus karinulus* Smith**
7	Hind coxa entirely smooth, polished	***Pristaulacus memnonius* Sun & Sheng**
–	Hind coxa transverse-carinate or rugose	**8**
8	Hind coxa rugose	***Pristaulacus zhejiangensis* He & Ma**
–	Hind coxa transverse-carinate	**9**
9	Forewing with vein 2-rs+m short, cells SM2 and D1 continuous	***Pristaulacus albitarsatus* Sun & Sheng**
–	Forewing with vein 2-rs+m long, cells SM2 and D1 distinctly separated (Figs 95, 118)	**10**
10	Metasoma entirely black (Fig. 98); hind tarsus black (Fig. 88)	***Pristaulacus obscurus* sp. n.**
–	Metasoma at least with first tergite brown (Figs 65, 121); hind tarsus yellowish-brown (Figs 56, 111)	**11**
11	Frons with yellow areas around antennae (Fig. 58); propleuron largely finely rugose with small smooth area posterodorsally (Fig. 60)	***Pristaulacus fopingi* sp. n.**
–	Frons entirely black (Fig. 113); propleuron densely punctate ventrally, finely rugose with small smooth area dorsally (Fig. 115)	***Pristaulacus rufobalteatus* Cameron**
12	Pronotum, in lateral view, with two projecting tooth-like processes, one anteroventral, the other ventral; pretarsal claw with six tooth-like processes	**13**
–	Pronotum with one anteroventrally projecting tooth-like process; pretarsal claw with four or five tooth-like processes	**16**
13	Occipital groove pronounced, as deep or deeper than wide	***Pristaulacus asiaticus* Turrisi & Smith**
–	Occipital groove shallow, less deep than wide (Fig. 103)	**14**
14	Ovipositor 1.4 × forewing length	***Pristaulacus nobilei* Turrisi & Smith**
–	Ovipositor 0.8–0.9 × forewing length	**15**
15	Forewing with vein 2-rs+m short, cells SM2 and D1 slightly separated; propleuron dull, finely rugose with small smooth area dorsally	***Pristaulacus iosephi* Turrisi & Madl**
–	Forewing with vein 2-rs+m long, cells SM2 and D1 distantly separated (Fig. 107); propleuron shiny, largely smooth with sparse fine punctures (Fig. 104)	***Pristaulacus pseudoiosephi* sp. n.**
16	Occipital carina not interrupted along occipital medial groove (Fig. 49); occipital medial groove V-shaped, its depth very shallow (Fig. 49)	***Pristaulacus excisus* Turner**
–	Occipital carina interrupted along occipital medial groove; occipital medial groove abruptly shaped, from narrow to wide and deep (Figs 27, 39)	**17**
17	Forewing with wide and irregular brown spots on basal part, below stigma and on apex	***Pristaulacus comptipennis* Enderlein**
–	Forewing with only one brown spot below stigma	**18**
18	Mesoscutum mostly areolate-rugose	***Pristaulacus porcatus* Sun & Sheng**
–	Mesoscutum mostly transverse-carinate (Figs 18, 29)	**19**
19	Setae on body golden brown (Fig. 24); punctures on frons deep and dense, distance between punctures 0.5–1.0 × diameter of a puncture (Fig. 26)	***Pristaulacus centralis* sp. n.**
–	Setae on body white (Fig. 13); punctures on frons deep and scattered, distance between punctures 2.0–3.0 × diameter of a puncture (Fig. 15)	***Pristaulacus calidus* sp. n.**

#### Pristaulacus albitarsatus

Taxon classificationAnimaliaHymenopteraAulacidae

Sun & Sheng, 2007

Fig. 122

Pristaulacus albitarsatus Sun & Sheng, 2007a: 216.
Pristaulacus albitarsatus Sun & Sheng: Turrisi et al. 2009: 56.

##### Material examined.

No available material for this study.

##### Diagnosis.

Metasoma more or less extensively reddish; hind tarsus withish-yellow; hind margin of head straight or weakly concave, without medial groove; occipital carina at most 0.2 × diameter of ocellus, pad-shaped, blackish; lateroventral margin of pronotum at least with one tooth-like process; forewing with vein 2-rs+m short, cells SM2 and D1 continuous; hind coxa transverse-carinate; hind basitarsus at most 1.3 × longer than tarsomeres 2–5 (Sun and Sheng 2007a).

##### Distribution.

China (Henan) (Sun and Sheng 2007a).

##### Biology.

Collected in May. Host not known (Sun and Sheng 2007a).

##### Remarks.

Unfortunately, we were unable to examine Sun & Sheng’s specimens. The diagnosis is based on the original description of Sun and Sheng (2007a).

#### Pristaulacus asiaticus

Taxon classificationAnimaliaHymenopteraAulacidae

Turrisi & Smith, 2011

Fig. 122

Pristaulacus asiaticus Turrisi & Smith, 2011: 10.

##### Material examined.

Holotype, ♂ (CAS), CHINA: W. Hupeh Prov., Lichuan District, Hsiao-Ho/10 August 1948, Gressit & Djou, Calif. Acad. Sciences/*Pristaulacus asiaticus* Turrisi & Smith sp. n.

##### Diagnosis.

Antenna with A1 dark reddish-brown on ventral surface; forewing infuscate, strongly infuscate on basal third and largely below stigma; hind margin of head grooved medially, occipital groove pronounced, as deep or deeper than wide; pronotum, in lateral view, with two projecting tooth-like processes, one anteroventral, the other ventral; pretarsal claw with six tooth-like processes; forewing with vein 2-rs+m long, cells SM2 and D1 distantly separated.

##### Distribution.

China (Hubei) (Turrisi and Smith 2011).

##### Biology.

Collected in August. Host not known (Turrisi and Smith 2011).

#### Pristaulacus calidus sp. n.

Taxon classificationAnimaliaHymenopteraAulacidae

http://zoobank.org/A4F26A90-5202-4075-B32F-9508528E8565

Figs 13 , 14–19 , 20–23 , 122

##### Material examined.

Holotype, ♂ (IZCAS), CHINA: Yunnan, Cheli, 560 m, 26.IV.1957, Da-hua Liu, IOZ(E) 1903971.

##### Etymology.

From the Latin adjective “*calidus*”, meaning “hot”, a noun in apposition.

##### Diagnosis.

Antenna black with scape yellowish-orange; metasoma black with posterior margin of first tergite brown; forewing hyaline with a small dark brown spot under stigma; occipital margin concave, with a wide and deep medial groove; pronotum with one anteroventrally projecting tooth-like process; pretarsal claw with five tooth-like processes; forewing with vein 2-rs+m short, cells SM2 and D1 slightly separated.

##### Description.

Holotype. *Male*. Body length 12.1 mm; forewing length 7.1 mm.

*Colour*. Antenna black with scape yellowish-orange; head black with clypeus dark brown; mesosoma black; metasoma black with posterior margin of first tergite brown; mandible brown with teeth darker; palpi black; fore leg, tibia and tarsus of mind leg and tarsus of hind leg yellowish-brown, remainder of legs dark brown to black; forewing hyaline with a small dark brown spot under stigma; hind wing hyaline.

*Head*. From above, 1.2 × wider than long, shiny; lower interocular distance 1.4 × eye height; malar space 0.4 × eye height; occipital margin concave, with a wide and deep medial groove; temple, from above, rounded, distinctly longer than eye length; occipital carina 0.5 × diameter of an ocellus; POL:OOL=0.9; lower frons and clypeus densely and finely punctate, remainder of head largely smooth with sparse and fine punctures; A3 3.5 × longer than wide; A4 5.8 × longer than wide, and 2.0 × longer than A3; A5 5.6 × longer than wide, and 1.9 × longer than A3.

*Mesosoma*. Pronotum coarsely areolate-rugose, with one anterior short tooth-like process on lateroventral margin; propleuron shiny and smooth ventrally, finely rugose on dorsal surface; mesoscutum mostly transverse-carinate, coarsely areolate-rugose along transscutal fissure and on sides, anterior part slightly emarginate medially, rounded laterally; notauli deep and wide; scutellum transverse-carinate in middle, coarsely areolate-rugose on anterior and posterior margin; axillae, metanotum, propodeum, mesopleuron and metapleuron coarsely areolate-rugose; forewing with vein 2-rs+m short, cells SM2 and D1 slightly separated; hind wing veins faint to absent; hind coxa transverse-carinate; hind basitarsus 10.0 × longer than wide, and 1.3 × of tarsomeres 2–5; pretarsal claw with five tooth-like processes.

*Metasoma*. Smooth, shining, with fine white pubescence on segment 2 to apex; petiole elongate, 5.7 × longer than wide.

Female. Unknown.

##### Distribution.

China (Yunnan).

##### Biology.

Collected in April. Host not known.

#### Pristaulacus centralis sp. n.

Taxon classificationAnimaliaHymenopteraAulacidae

http://zoobank.org/0B488BA3-8A8A-4C82-AF41-7F07BB80C214

Figs 24 , 25–30 , 31–34 , 122

##### Material examined.

Holotype, ♀ (IZCAS), CHINA: Hubei, Zigui, Mt. Jiulingtou, 250 m, 27.VII.1993, Xiao-lin Chen, IOZ(E) 1903961.

##### Etymology.

From the Latin adjective “*centralis*”, meaning “placed in the middle”, a noun in apposition.

##### Diagnosis.

Forewing with only one brown spot below stigma; metasoma mostly yellowish-brown with first tergite largely black; occipital margin concave, with a strongly wide and deep medial groove; pronotum with one anteroventrally projecting tooth-like process; mesoscutum mostly transverse-carinate; pretarsal claw with four tooth-like processes; ovipositor 1.6 × forewing length.

##### Description.

Holotype. *Female*. Body length 14.2 mm; forewing length 9.4 mm.

*Colour*. Antenna black with scape yellowish-orange; head black with clypeus orange; mesosoma black; metasoma mostly yellowish-brown with first tergite largely black; mandible orange with teeth dark brown; palpi dark brown; hind coxa black, remainder of legs yellowish-orange with tarsi paler; ovipositor sheath black; forewing infuscate, with dark brown spot under stigma; hind wing hyaline.

*Head*. From above, 1.3 × wider than long, shiny; lower interocular distance 1.4 × eye height; malar space 0.4 × eye height; occipital margin concave, with a strongly wide and deep medial groove; temple, from above, rounded, slightly shorter than eye length; occipital carina 0.8 × diameter of an ocellus; POL:OOL=1.1; frons, clypeus and marlar space densely and finely punctate; vertex and temple largely smooth with sparse and fine punctures; A3 6.5 × longer than wide; A4 8.7 × longer than wide, and 1.3 × longer than A3; A5 9.6 × longer than wide, and 1.1 × longer than A3.

*Mesosoma*. Pronotum coarsely areolate-rugose, with one anterior short tooth-like process on lateroventral margin; propleuron dull, largely finely rugose or punctate with small smooth area posterodorsally; mesoscutum mostly transverse-carinate, coarsely rugose on sides, anterior part slightly emarginate medially, rounded laterally; notauli deep and wide; scutellum transverse-carinate in middle, coarsely rugose on posterior margin; axillae, metanotum and propodeum coarsely areolate-rugose; mesopleuron mostly coarsely areolate-rugose with small rugose area anteriodorsally; metapleuron coarsely areolate-rugose; forewing with vein 2-rs+m short, cells SM2 and D1 slightly separated; hind wing with veins somewhat distinct, cells Cu and R1+Rs contiguous; hind coxa transverse-carinate; hind basitarsus 9.5 × longer than wide, and 1.2 × of tarsomeres 2–5; pretarsal claw with four tooth-like processes.

*Metasoma*. Smooth, shining, with fine white pubescence on segment 3 to apex; petiole elongate, slender, 4.5 × longer than wide; ovipositor 1.6 × forewing length.

Male. Unknown.

##### Distribution.

China (Hubei).

##### Biology.

Collected in July. Host not known.

#### Pristaulacus comptipennis

Taxon classificationAnimaliaHymenopteraAulacidae

Enderlein, 1912

Figs 35 , 36–42 , 43–44 , 122

Pristaulacus comptipennis Enderlein, 1912: 265.
Pristaulacus comptipennis Enderlein: Enderlein 1913: 319, 326; Hedicke 1939: 7; Konishi 1990: 652; 1991: 564; Smith 2001: 282; Turrisi 2007: 28; Turrisi et al. 2009: 56; Turrisi and Smith 2011: 14.

##### Material examined.

Lectotype, ♀ (SDEI), TAIWAN: Hoozan, Formosa, II.10, H. Sauter/*Pristaulacus comptipennis* Enderl., ♀, Type, Dr. Enderlein det. 1912/Syntypus/Eberswalde coll. DEI/Lectotypus ♀, *Pristaulacus comptipennis* Enderlein, 1912, des. T. Megjaszai 1999/*Pristaulacus comptipennis* Enderlein, 1912, ♀, Lectotypus G.F. Turrisi des. 2006. Paralectotypes: 2 ♀♀ (SDEI), Hoozan, Formosa, V.10, H. Sauter/*Pristaulacus comptipennis* Enderl., ♀, Type, Dr. Enderlein det. 1912/Syntypus/Eberswalde coll. DEI/Paralectotypus ♀, *Pristaulacus comptipennis* Enderlein, 1912, des. T. Megjaszai 1999/*Pristaulacus comptipennis* Enderlein, 1912, ♀, Paralectotypus G.F. Turrisi des. 2006. Additional material: 1 ♀ (SDEI), Taiwan, Hoozan, V.1910, H. Sauter; 2 ♀♀ (SDEI), Taiwan, Anping, 22.VII.1911, H. Sauter; 1 ♀ (SDEI), Taiwan, Kankau (Koshun), V.1912, H. Sauter; 2 ♀♀ (SDEI, USNM), Taiwan, Kosempo, H. Sauter; 1 ♀ (SDEI), Taiwan, Kosempo, 1911, H. Sauter; 2 ♂♂ (SDEI, USNM), Taiwan, Kosempo, 1912, H. Sauter; 7 ♂♂ (SDEI), Taiwan, Kosempo, V.1912, H. Sauter; 2 ♀♀, 2 ♂♂ (SDEI), Taiwan, Tainan, 22.VII.1911, H. Sauter; 2 ♀♀ (SDEI), Taiwan, Taihorin, V.1910, H. Sauter; 1 ♀ (ZMHB), Taiwan, Hoozan, IX.1910, Sauter (ZMHB); 2 ♀♀ (ZMHB), Taiwan, Taihorish, VI.1910, H. Sauter S-G.; 1 ♀, 1 ♂ (ZMHB), Taiwan, IX.1910; 1 ♂ (ZMHB), Taiwan, X.1910; 1 ♀ (HNHM), Taiwan, Taihorinsho, IX.1909, Sauter; 3 ♀♀ (HNHM), Taiwan, Kosempo, IX.1909, Sauter; 1 ♀ (LACM), Taiwan, Puli Village, Nam-tou, Hsien, 15-30.XII.1963, coll. K.H. Chen. 1 ♀ (NHRS) Taiwan, Hoozan, 7.IX.1910, H. Sauter leg. (labelled as “Cotypus”); 1 ♂ (USNM), Taiwan, Keelung, 1910, Victor Kühne leg. CHINA: 1 ♀ (IZCAS), Hainan, Mt. Jianfengling, 2.VI.1982, Pei-zheng Chen, IOZ(E) 1903947; 1 ♀ (SCAU), Hunan, Yongzhou, 27.VI.1981, Tong Xin-wang; 1 ♀ (USNM), Hong Kong, Pak Sha O, 22.25N, 114.19E, 3.VI.2005, Ch. Bartelemy leg.; 1 ♀ (TCUC), Hong Kong, Tai Po Kau Forest, 50Q KK 094 813, 370 m, 21.VI.2006, Ch. Barthélémy leg.

##### Diagnosis.

Antenna black with scape brown; forewing with wide and irregular brown spots on basal part, below stigma and on apex; metasoma black with second tergite brown anteriorly; occipital margin concave, with a wide and deep medial groove; pronotum with one anteroventrally projecting tooth-like process; ovipositor 1.2 × forewing length.

##### Distribution.

China (Taiwan, Hunan, Hongkong, Hainan); Korea; Japan; Laos (Turrisi and Smith 2011; Choi et al. 2013).

##### Remarks.

Redescriptions and data on intraspecific variation are provided by Konishi (1990, 1991) and Turrisi (2007). Additional notes on identification and distribution are provided by Turrisi and Smith (2011).

##### Biology.

Collected in May–July, September, October, and December. Host: *Ceresium elongatum* Matsushita, 1933 (Coleoptera, Cerambycidae) (Konishi 1991) and *Olenecamptus bilobus nipponensis* Dillon & Dillon (Coleoptera, Cerambycidae) (Turrisi and Smith 2011).

#### Pristaulacus excisus

Taxon classificationAnimaliaHymenopteraAulacidae

Turner, 1922

Figs 45 , 46–51 , 52–55 , 122

Pristaulacus excisus Turner 1922: 271.
Pristaulacus excisus Turner: Hedicke 1939: 7; Smith 2001: 283; Turrisi et al. 2009: 57; Turrisi and Smith 2011: 25.

##### Material examined.

Holotype, ♀ (BMNH) examined (see Turrisi and Smith 2011). Additional material: 1 ♂ (IZCAS), CHINA: Guangxi, Ningming, 102 m, 17.V.1984, Gui-biao Luo, IOZ(E) 1903963; 1 ♂ (IZCAS), Guangxi, Chongming, 110 m, 20.V.1984, Jin-yi Huang, IOZ(E) 1903964.

##### Diagnosis.

Metasoma black with transverse patch near posterior margin of first tergite and anterior margin of second tergite yellowish-brown; forewing infuscate, with anterior third darker and dark brown spot under stigma; occipital margin concave, V-shaped, its depth very shallow; pronotum with one anteroventrally projecting tooth-like process; pretarsal claw with four tooth-like processes.

##### Distribution.

China (Guangxi); Vietnam (Turrisi and Smith 2011).

##### Biology.

Collected in May and August (Turrisi and Smith 2011). Host not known.

##### Remarks.

Redescription is provided by Turrisi (2007). This is the first description of the male and the first record of this species from China.

#### Pristaulacus fopingi sp. n.

Taxon classificationAnimaliaHymenopteraAulacidae

http://zoobank.org/68C2B82D-9CEB-48BD-9097-DEF7A9CE4E2A

Figs 56 , 57–62 , 63–65 , 122

##### Material examined.

Holotype, ♂ (IZCAS), CHINA: Shaanxi, Foping, 900 m, 27.VI.1999, Jian Hu, IOZ(E) 1903962.

##### Etymology.

Named after the type locality.

##### Diagnosis.

Frons with yellow areas around antennae; hind margin of head straight, without medial groove; lateroventral margin of pronotum with one tooth-like process; propleuron largely finely rugose with small smooth area posterodorsally; forewing with vein 2-rs+m long, cells SM2 and D1 distantly separated; hind coxa transverse-carinate.

##### Description.

Holotype. *Male*. Body length 12.3 mm; forewing length 8.8 mm.

*Colour*. Antenna black with scape brown; head black with clypeus and lower frons under antennal sockets yellow; mesosoma black; metasoma black with first tergite and anterior margin of second tergite brown; mandible dark brown; palpi yellowish-brown; coxae and hind femura black, remainder of legs yellowish-orange with tarsi paler; forewing slightly infuscate, with dark brown spot under stigma; hind wing hyaline.

*Head*. From above, 1.2 × wider than long, shiny; lower interocular distance 1.5 × eye height; malar space 0.3 × eye height; occipital margin straight; temple, from above, rounded, slightly shorter than eye length; occipital carina 0.1 × diameter of an ocellus; POL:OOL=0.8; frons above antennal sockets and marlar space densely and finely punctate, remainder of head largely smooth with sparse and fine punctures; A3 3.0 × longer than wide; A4 5.0 × longer than wide, and 1.6 × longer than A3; A5 5.4 × longer than wide, and 1.7 × longer than A3.

*Mesosoma*. Pronotum coarsely areolate-rugose, with one anterior short tooth-like process on each lateroventral margin; propleuron dull, largely finely rugose with small smooth area posterodorsally; mesoscutum transverse-carinate anteriorly, coarsely areolate-rugose posterior to notauli, anterior part emarginate medially, rounded laterally; notauli deep and wide posteriorly, becoming narrower anteriorly; scutellum transverse-carinate in middle, coarsely rugose on posterior margin; axillae, metanotum and propodeum coarsely areolate-rugose; mesopleuron mostly coarsely areolate-rugose with small rugose area anteriodorsally; metapleuron coarsely areolate-rugose; forewing with vein 2-rs+m long, cells SM2 and D1 distantly separated; hind wing with veins distinct, cells Cu and R1+Rs contiguous; hind coxa transverse-carinate; hind basitarsus 9.6 × longer than wide, and 1.2 × of tarsomeres 2–5; pretarsal claw with four tooth-like processes.

*Metasoma*. Smooth, shining, with fine white pubescence on segment 3 to apex; petiole elongate, slender, 3.5 × longer than wide.

Female. Unknown.

##### Distribution.

China (Shaanxi).

##### Biology.

Collected in June. Host not known.

#### Pristaulacus intermedius

Taxon classificationAnimaliaHymenopteraAulacidae

Uchida, 1932

Figs 66 , 67–73 , 74–75 , 122

Pristaulacus intermedius Uchida, 1932: 190.
Pristaulacus intermedius Uchida: Hedicke 1939: 11; Smith 2001: 288; Turrisi 2007: 48; Lee and Turrisi 2008: 115; Turrisi et al. 2009: 57; Smith and Tripotin 2011: 523.

##### Material examined.

CHINA: 1 ♀ (IZCAS), Jilin, Jiaogou, 21.VII.1985, IOZ(E) 1903967; 1 ♀ (IZCAS), Jilin, Mangjiang, 26.VII.1955, Zhi-yin Li, IOZ(E) 1903965; 1 ♀ (IZCAS); Yunnan, Menghai, 17.IV.1982, Chun-mei Huang, IOZ(E) 1903966; 1 ♀ (OLML), Shaanxi province, Mounts Qinling, Xunyangba (6 km E), 1000–1300 m, 23.V–13.VI.1998, I.H. Marshal leg.

##### Diagnosis.

Forewing slightly infuscate, with large dark brown spot under stigma; metasoma entirely blackish, at most slightly lightened basally; basal antennomeres elongate (A3 5.0–6.0 × longer than wide, A4 10.0 × longer than wide); occipital margin straight, wide, 0.5 × diameter of ocellus, lamelliform, brownish; lateroventral margin of pronotum without process; ovipositor 1.3 × forewing length.

##### Distribution.

China (Liaoning, Jilin, Shaanxi, Yunnan); Japan; South Korea (Lee and Turrisi 2008; Smith and Tripotin 2011).

##### Biology.

Collected from April to August. Host: *Chlorophorus japonicus* (Chevrolat, 1863) (Coleoptera, Cerambycidae) (Uchida 1932).

##### Remarks.

This is the first record of this species from the Oriental Region.

#### Pristaulacus iosephi

Taxon classificationAnimaliaHymenopteraAulacidae

Turrisi & Madl, 2013

Figs 76 , 77–82 , 83–87 , 122

Pristaulacus iosephi Turrisi & Madl, 2013: 239.

##### Material examined.

Holotype, ♀ (BPBM), THAILAND: NW. Chiangmai: Fang, 500 m. IV–12–19–’58/T.C. Maa Collector, No. 388/*Pristaulacus iosephi* Turrisi and Madl sp. n. ♀ 2010 Holotypus. Additional material: CHINA: 1 ♀ (IZCAS), Yunnan, Baoshan, 1700 m, 18.V.1955, Kpыжановский O. Ӆ., IOZ(E) 1903954.

##### Diagnosis.

Metasoma black with posterior half of first tergite brown; forewing infuscate, with anterior third darker and large dark brown spot under stigma; lateral margin of pronotum with two well-developed tooth-like processes; pretarsal claw with six tooth-like processes; ovipositor 0.8 × forewing length.

##### Distribution.

China (Yunnan); Thailand (Turrisi and Madl 2013).

##### Biology.

Collected in April and May. Host not known.

##### Remarks.

This species is newly recorded from China.

#### Pristaulacus karinulus

Taxon classificationAnimaliaHymenopteraAulacidae

Smith, 2001

Fig. 122

Pristaulacus kiefferi Enderlein, 1912: 266 (preoccupied by Bradley 1908).
Pristaulacus karinulus Smith, 2001: 288 (replacement name for Pristaulacus kiefferi Enderlein, 1912).
Pristaulacus karinulus Smith: Sun and Sheng 2007a: 219; Turrisi et al. 2009: 57.

##### Material examined.

CHINA: 1 ♀, Taiwan (Hoozan), labelled as syntypus of *Pristaulacus kiefferi* (SDEI).

##### Diagnosis.

Metasoma extensively reddish orange; basal antennomeres elongate (A3 5.0–6.0 × longer than wide, A4 10.0 × longer than wide); occipital carina straight, wide, 0.5 × diameter of ocellus, lamelliform, brownish.

##### Distribution.

China (Henan, Jiangsu, Taiwan) (Sun and Sheng 2007a); India (Smith 2001).

##### Biology.

Collected from May to July. Host not known.

##### Remarks.

Sun and Sheng (2007a) recorded this species from Henan and Jiansu. However, we were unable to examine Sun & Sheng’s specimens.

#### Pristaulacus longicornis

Taxon classificationAnimaliaHymenopteraAulacidae

Kieffer, 1911

Pristaulacus longicornis Kieffer, 1911: 230.
Pristaulacus longicornis Kieffer: Kieffer 1912: 386; Hedicke 1939: 12; Smith 2001: 289; Turrisi 2007: 54; Turrisi et al. 2009: 57.

##### Material examined.

Holotype, ♀, CHINA: “B.M. Type Hym. 3.a.99/*Pristaulacus longicornis* Kieff./F. Sm. Coll. 79.22/ determined by Dr. Kieffer” (BMNH).

##### Diagnosis.

Mandible extensively dark red, with base and apex blackish; forewing slightly infuscate at apex, with a small irregular and narrow brown substigmal spot and a small irregular brown spot on middle part of B; metasoma reddish-brown, with T1 and T2 extensively reddish-orange and petiole blackish; occipital carina wide, lamelliform, 0.5 × diameter of an ocellus; A3 8.3 × longer than wide; A4 14.0 × longer than wide, and 1.7 × longer than A3; pronotum with a weak anterior tooth-like process on lateroventral margin; hind basitarsus 14.0 × longer than wide, and slightly longer than tarsomeres 2–5; pretarsal claw with four tooth-like processes; metasoma with petiole elongate and slender, 2.0 × longer than wide.

##### Distribution.

China (unknown whether Palaearctic or Oriental) (Turrisi 2007; Turrisi et al. 2009).

##### Biology.

Unknown.

##### Remarks.

Redescription is provided by Turrisi (2007).

#### Pristaulacus memnonius

Taxon classificationAnimaliaHymenopteraAulacidae

Sun & Sheng, 2007

Fig. 122

Pristaulacus memnonius Sun & Sheng, 2007a: 217.
Pristaulacus memnonius Sun & Sheng: Turrisi et al. 2009: 57.

##### Material examined.

Paratype: 1 ♀ (SFPS), CHINA: Lingshan, Henan, 1999.5.24, M.-L. Sheng//400–500 m, 1999.5.24/*Pristaulacus memnonius* Sun & Sheng.

##### Diagnosis.

Hind margin of head straight; occipital carina about 0.2 × diameter of ocellus, pad-shaped, blackish; lateroventral margin of pronotum with one tooth-like process; hind coxa entirely smooth, polished.

##### Distribution.

China (Henan) (Sun and Sheng 2007a).

##### Biology.

Collected in May (Sun and Sheng 2007a). Host not known.

#### Pristaulacus nobilei

Taxon classificationAnimaliaHymenopteraAulacidae

Turrisi & Smith, 2011

Fig. 122

Pristaulacus nobilei Turrisi & Smith, 2011: 41.

##### Material examined.

Holotype, ♀ (ZMHB), CHINA: Canton (China), Westfluss, Ting-Wu-San, Mell S.G./Zool. Mus. Berlin/[unreadable handwritten label]/*Pristaulacus nobilei* Turrisi & Smith sp. n., ♀, 2009, Holotypus. Paratypes: 1 ♀ (USNM), F, China, NGistGee, coll./*Pristaulacus nobilei* Turrisi & Smith sp. n., ♀, 2009, Paratypus; 3 ♀♀ (BMNH), China Macao/*Pristaulacus nobilei* Turrisi & Smith sp. n., ♀, 2009, Paratypus; 1 ♀ (USNM), Tai-o Lantau Isl, Hong Kong, VI.12.1978/RD Montgomery colr/Davis/USNM 2046975/*Pristaulacus nobilei* Turrisi & Smith sp. n., ♀, 2009, Paratypus.

##### Diagnosis.

Metasoma with second tergite extensively dark reddish; occipital margin weakly grooved medially; lateroventral margin of pronotum with two well-developed tooth-like processes; pretarsal claw with six tooth-like processes; ovipositor 1.4 × forewing length.

##### Distribution.

China (Jiangsu, Guangdong, Hongkong, Macao) (Turrisi and Smith 2011).

##### Biology.

Collected in June. Host not known (Turrisi and Smith 2011).

#### Pristaulacus obscurus sp. n.

Taxon classificationAnimaliaHymenopteraAulacidae

http://zoobank.org/65606CFD-531D-4BD4-9AED-FB05CC4976C5

Figs 88 , 89–94 , 95–98 , 122

##### Material examined.

Holotype, ♀ (IZCAS), CHINA: Yunnan, Nanhua County, 2400 m, 24.VI.1980, Pei-zhi Yang, IOZ(E) 1903948. Paratype, 1 ♀ (IZCAS), CHINA: Yunnan, Jingdong, Waidaba, 1250 m, 26.V.1956, Xing-chi Yang, IOZ(E) 1903949.

##### Etymology.

From the Latin adjective “*obscurus*”, meaning “dark, black”, a noun in apposition.

##### Diagnosis.

Body and legs entirely black; forewing hyaline, with dark brown spot under stigma; occipital margin straight; lateroventral margin of pronotum with one tooth-like processes; forewing with vein 2-rs+m long, cells SM2 and D1 distantly separated; ovipositor 0.8 × forewing length.

##### Description.

Holotype. *Female*. Body length 12.0 mm; forewing length 9.4 mm.

*Colour*. Black except: forewing hyaline, with dark brown spot under stigma; hind wing hyaline.

*Head*. From above, 1.3 × wider than long, shiny; lower interocular distance 1.7 × eye height; malar space 0.3 × eye height; occipital margin straight; temple, from above, rounded, distinctly shorter than eye length; occipital carina 0.1 × diameter of an ocellus; POL:OOL=1.0; head largely smooth except frons above and lateral antenna densely and finely punctate; A3 3.7 × longer than wide; A4 6.0 × longer than wide, and 1.6 × longer than A3; A5 6.3 × longer than wide, and 1.8 × longer than A3.

*Mesosoma*. Pronotum coarsely areolate-rugose, with one anterior short tooth-like process on lateroventral margin; propleuron smooth and shiny; mesoscutum transverse-carinate anteriorly, irregularly rugose posterior to notauli, anterior part strongly emarginate medially, slightly pointed laterally; notauli deep and wide; scutellum transverse-carinate medially, areolate-rugose laterally; axillae areolate-rugose; metanotum, propodeum, mesopleuron and metapleuron coarsely areolate-rugose; forewing with vein 2-rs+m long, cells SM2 and D1 distantly separated; hind wing veins faint to absent; hind coxa transverse-carinate; hind basitarsus 8.5 × longer than wide, and 1.2 × of tarsomeres 2–5; pretarsal claw with four tooth-like processes.

*Metasoma*. Smooth, shining, with fine white pubescence on segment 3 to apex; petiole elongate, 3.8 × longer than wide; ovipositor 0.8 × forewing length.

Male. Unknown.

##### Distribution.

China (Yunnan).

##### Biology.

Collected in May and June. Host not known.

#### Pristaulacus pieli

Taxon classificationAnimaliaHymenopteraAulacidae

Kieffer, 1924

Fig. 122

Pristaulacus pieli Kieffer, 1924: 79.
Pristaulacus pieli Kieffer: Hedicke 1939: 14; Wu 1941: 90; Smith 2001: 294; Turrisi et al. 2009: 58.

##### Material examined.

The type material is not known (Smith 2001), and no additional specimen is currently known.

##### Diagnosis.

Hind margin of head straight, without medial groove; lateroventral margin of pronotum without tooth-like process (Kieffer 1924).

##### Distribution.

China (Jiangsu).

##### Biology.

The holotype was collected in July. Host not known.

#### Pristaulacus porcatus

Taxon classificationAnimaliaHymenopteraAulacidae

Sun & Sheng, 2007

Fig. 122

Pristaulacus porcatus Sun & Sheng, 2007a: 217.
Pristaulacus porcatus Sun & Sheng: Turrisi et al. 2009: 58; Turrisi and Smith 2011: 43.

##### Material examined.

Paratype, 1 ♀ (SFPS), CHINA: Henan, Lingshan, 400–500 m, 24.V.1999, M. L. Sheng/*Pristaulacus porcatus* Sun & Sheng, sp. n.

##### Diagnosis.

Antenna with A1 light orange and A2 dark reddish; metasoma with side of first tergite, most of second tergite and side of third tergite irregulary orange; occipital margin concave, with a wide and deep medial groove; pronotum with one anteroventrally projecting tooth-like process; pretarsal claw with four tooth-like processes; ovipositor 1.2 × forewing length.

##### Distribution.

China (Henan) (Sun and Sheng 2007a).

##### Biology.

Collected in May (Sun and Sheng 2007a). Host not known.

##### Remarks.

Redescription is provided by Turrisi and Smith (2011).

#### Pristaulacus pseudoiosephi sp. n.

Taxon classificationAnimaliaHymenopteraAulacidae

http://zoobank.org/B66E7537-6642-4B01-9DE2-7315591CDC6F

Figs 99 , 100–106 , 107–110 , 122

##### Material examined.

Holotype, ♀ (IZCAS), CHINA: Guangxi, Longteng, Mt. Tianping, 740 m, 18.VI.1962, Shu-yong Wang, IOZ(E) 1903953. Paratypes: 1 ♀ (IZCAS), CHINA: Yunnan, Jinping, Mengla, 420 m, 21.IV.1956, Ke-ren Huang et al., IOZ(E) 1903951; 1 ♀ (IZCAS), Yunnan, Jinping, Mengla, 400 m, 24.IV.1956, Ke-ren Huang et al., IOZ(E) 1903952.

##### Etymology.

The name refers to the similar appearance to *Pristaulacus iosephi*.

##### Diagnosis.

Body black; forewing infuscate, with large dark brown spot under stigma; occipital margin concave, with a wide and deep medial groove; pronotum with two anteroventrally projecting tooth-like process; pretarsal claw with six tooth-like processes; ovipositor 0.9 × forewing length.

##### Description.

Holotype. *Female*. Body length 16.6 mm; forewing length 11.3 mm.

*Colour*. Black except: scape of antenna, palpi and tarsi dark brown; forewing infuscate, with large dark brown spot under stigma; basal 2/3 of hind wing hyaline, apical 1/3 infuscate.

*Head*. From above, 1.2 × wider than long, shiny; lower interocular distance 1.3 × eye height; malar space 0.2 × eye height; occipital margin concave, with a wide and deep medial groove; temple, from above, rounded, distinctly longer than eye length; occipital carina 0.3 × diameter of an ocellus; POL:OOL=1.1; frons and clypeus densely and finely punctate; vertex and temple largely smooth with sparse and fine punctures; A3 2.4 × longer than wide; A4 3.7 × longer than wide, and 1.8 × longer than A3; A5 3.2 × longer than wide, and 1.5 × longer than A3.

*Mesosoma*. Pronotum coarsely areolate-rugose, with two well-developed anterior and posterior tooth-like processes on lateroventral margin; propleuron largely smooth with sparse fine punctures, shiny; mesoscutum transverse-carinate anteromedially, remainder of mesocutum coarsely areolate-rugose, anterior part strongly emarginate medially, rounded laterally; notauli deep but narrow; scutellum, axillae, metanotum, propodeum, mesopleuron and metapleuron coarsely areolate-rugose; forewing with vein 2-rs+m long, cells SM2 and D1 distantly separated; hind wing with veins somewhat distinct, cells Cu and R1+Rs contiguous; hind coxa transverse-carinate; hind basitarsus 11.3 × longer than wide, and 1.1 × of tarsomeres 2–5; pretarsal claw with six tooth-like processes.

*Metasoma*. Smooth, shining, with fine white pubescence on segment 3 to apex; petiole elongate, 3.3 × longer than wide; ovipositor 0.9 × forewing length.

Male. Unknown.

##### Distribution.

China (Guangxi, Yunnan).

##### Biology.

Collected in April and June. Host not known.

#### Pristaulacus rufipes

Taxon classificationAnimaliaHymenopteraAulacidae

Enderlein, 1912

Fig. 122

Pristaulacus rufipes Enderlein, 1912: 266.
Pristaulacus rufipes Enderlein: Hedicke 1939: 15; Smith 2001: 295; Turrisi et al. 2009: 58.

##### Material examined.

Holotypus, ♀ (SDEI), CHINA: Formosa, Hoozan, Sauter H./*Pristaulacus rufipes* Enderlein sp. n. Other material: 1 ♂ (TCUC), Taiwan, Gaofong Ln., about 1400 m, Ren-ai T. Nantou, 7–9.V.2009, Takakuwa M. leg.

##### Diagnosis.

Antenna reddish-orange with A1 lighter; legs light reddish-orange, except coxae and hind trochanters darker; metasoma largely black, except S1, most part of T2 and apex of following tergites dark reddish; occipital margin straight; pronotum with one anteroventrally projecting tooth-like process; pretarsal claw with four tooth-like processes; ovipositor 1.8 × forewing length.

##### Redescription.

Holotype. *Female*. Body length 14.8 mm; forewing length 11.9 mm.

*Colour*. Blackish-brown except: clypeus extensively dark brown; mandible extensively reddish-orange, with apex blackish; maxillo-labial complex brownish to dark brownish; antenna reddish-orange with A1 lighter; legs light red orange, except coxae and hind trochanter darker; wings hyaline, forewing with a wide brown spot below stigma (two third as wide as stigma width) not extending beyond cells SM-1 and R; metasoma largely black, except S1, most part of T2 and apex of following tergites dark reddish; valvula 3 of ovipositor dark brown to blackish-brown. Setae: whitish to goldish.

*Head*. From above, 1.4 × wider than long, shiny; occipital margin straight; temple, from above, weakly developed, weakly convex; occipital carina about 0.2 × diameter of an ocellus; POL:OOL= 1.2; vertex and temple with fine, and scattered to dense punctures (distance between punctures 3.0–1.5 × diameter of a puncture); frons with coarse, and scattered to dense punctures (distance between punctures 3.0–1.0 × diameter of a puncture); clypeus with coarse, and dense punctures; malar area with coarse, and dense punctures; occipital area with fine, and dense punctures (distance between punctures about 1.5 × diameter of an ocellus).

*Mesosoma*. Coarsely sculptured; pronotum areolate punctate, except lower third, coarsely punctate to areolate rugulose, with one weakly developed anterior tooth on each lateroventral margin; propleuron polished and shiny, coarsely, deeply, and densely punctate-rugulose on dorsal surface, with coarse, deep, and scattered to dense punctures on ventral surface (distance between punctures 1.0–2.0 × diameter of a puncture); prescutum sub-triangular, very wide, not concave, transverse-carinulate-punctate to transverse-carinate; mesoscutum transverse-carinate, with anterior part slightly emarginate in middle, rounded (lateral view); notauli deep and narrow; scutellum transverse-carinate; mesopleuron areolate-rugose (upper part) to rugulose-punctate-carinulate (lower part), except a wide part of subalar area, punctate-rugulose; metanotum mostly smooth, with a few confused carinulae; propodeum areolate-rugose, except anterior margin longitudinally carinate; ventral parts of mesosoma rugose to punctate; forewing with vein 2-rs+m short, cells SM2 and D1 continuous; fore coxa polished with coarse, deep, and dense punctures; mid coxa rugulose-punctate; hind coxa with very coarse, deep, and dense punctures on most of dorsal surface (with a few transverse weakly defined carinae in middle), mostly polished (rugose on sides) and punctate on ventral surface (punctures coarse, deep, and dense, distances between punctures 0.5–1.0 × diameter of a puncture); hind basitarsus 13.4 × longer than wide and 1.9 × longer than tarsomeres 2–5; pretarsal claw with four tooth-like processes.

*Metasoma*. Pyriform (lateral view), compressed laterally; petiole elongate, slender, 4.4 × as long as wide; segments 1 and 2 polished and shiny; following segments with fine and dense punctures; ovipositor 1.8 × forewing length.

Male. Similar to the female, but metasoma darker.

##### Distribution.

China (Taiwan).

##### Biology.

Unknown.

#### Pristaulacus rufobalteatus

Taxon classificationAnimaliaHymenopteraAulacidae

Cameron, 1907

Figs 111 , 112–117 , 118–121 , 122

Pristaulacus rufobalteatus Cameron, 1907: 222.
Pristaulacus rufobalteatus Cameron: Smith 2001: 297; Turrisi et al. 2009: 58.

##### Material examined.

1 ♀ (IZCAS), CHINA: Yunnan, Jingdong, 1200 m, 6.III.1957, IOZ(E) 1903968; 1 ♀ (IZCAS), Yunnan, Lushui, 1900 m, 8.VI.1981, Su-bo Liao, IOZ(E) 1903969; 1 ♀ (IZCAS), CHINA: Gansu, Kang County, Qinghe Forestry Station, 2250 m, 8.VII.1999, Hong-jian Wang, IOZ(E) 1903970.

##### Diagnosis.

Metasoma black with first tergite largely brown; fore hyaline with dark brown spot under stigma; occipital margin straight; occipital carina 0.1 × diameter of an ocellus; pronotum with one anteroventrally projecting tooth-like process; pretarsal claw with four tooth-like processes; ovipositor 0.9 × forewing length.

##### Redescription.

*Female*. Body length 8.7 mm; forewing length 6.8 mm.

*Colour*. Antenna black with scape yellowish-orange; head black with clypeus dark brown; mesosoma black; first tergite largely brown, and remainder of tergites black; mandible brown with teeth darker; palpi brown; coxae black, femur and tibia dark brown, remainder of legs yellowish-orange with tarsi paler; ovipositor brown; fore hyaline with dark brown spot under stigma; hind wing hyaline.

*Head*. From above, 1.4 × wider than long, shiny; lower interocular distance 1.5 × eye height; malar space 0.3 × eye height; occipital margin straight; temple, from above, rounded, slightly shorter than eye length; occipital carina 0.1 × diameter of an ocellus; POL:OOL=1.3; lower frons and clypeus densely and finely punctate, remainder of head largely smooth with sparse and fine punctures; A3 3.7 × longer than wide; A4 6.6 × longer than wide, and 1.7 × longer than A3; A5 6.2 × longer than wide, and 1.5 × longer than A3.

*Mesosoma*. Pronotum coarsely rugose, with one anterior small process on lateroventral margin; propleuron dull, densely punctate ventrally, finely rugose with small smooth area dorsally; mesoscutum mostly transverse-carinate, coarsely rugose on sides, anterior part slightly emarginate medially, rounded laterally; notauli deep and wide; scutellum transverse-carinate in middle, coarsely rugose on anterior and posterior margin; axillae coarsely areolate-rugose; metanotum coarsely rugose; propodeum largely coarsely areolate-rugose, coarsely rugose in middle; mesopleuron coarsely areolate-rugose posteriodorsally, remainder rugose; metapleuron coarsely areolate-rugose; forewing with vein 2-rs+m long, cells SM2 and D1 distantly separated; hind wing veins faint to absent; hind coxa transverse-carinate; hind basitarsus 8.5 × longer than wide, and 1.3 × of tarsomeres 2–5; pretarsal claw with four tooth-like processes.

*Metasoma*. Smooth, shining, with fine white pubescence on segment 2 to apex; petiole elongate, 2.4 × longer than wide; ovipositor 0.9 × forewing length.

Male. Unknown.

##### Distribution.

China (Gansu, Yunnan); India (Cameron 1907).

##### Biology.

Collected in March, June and July. Host not known.

##### Remarks.

This is a newly recorded species for China.

#### Pristaulacus zhejiangensis

Taxon classificationAnimaliaHymenopteraAulacidae

He & Ma, 2002

Fig. 122

Pristaulacus zhejiangensis He & Ma, 2002: 150.
Pristaulacus zhejiangensis He & Ma: Turrisi et al. 2009: 59.

##### Material examined.

Holotype, ♀ (ZJU), CHINA: Zhejiang, Mt. Fengyang, 16.VIII.1982, De-ming Yu, No. 826802/*Pristaulacus zhejiangensis* He & Ma, sp. n. Paratypes: 1 ♀ (ZJU), Zhejiang, Mt. Fengyang, 19.IV.1984, li-rong Shen, No. 843798/*Pristaulacus zhejiangensis* He & Ma, sp. n. Additional material: 1 ♀ (SEMC), CHINA: Fujian, Yong’an, Xiyang, 27.IV.1960, Geng-tao Jin, 34008303; 1 ♀ (SCAU), CHINA: Hunan, 1981, Tong Xin-wang.

##### Diagnosis.

Metasoma black with posterior margin of first tergite dark brown; tibiae and tarsi yellowish-brown with tarsi paler, remainder of legs dark brown to black; forewing infuscate, with large dark brown spot under stigma; occipital margin straight; occipital carina 0.1 × diameter of an ocellus; pronotum with one anteroventrally projecting tooth-like process; pretarsal claw with four tooth-like processes; ovipositor 0.9 × forewing length.

##### Redescription.

*Female*. Body length 10.1 mm; forewing length 7.2 mm.

*Colour*. Black except: scape of antenna dark brown; mandible yellowish-brown with teeth darker; posterior margin of first tergite dark brown; tibiae and tarsi yellowish-brown with tarsi paler, remainder of legs dark brown to black; forewing infuscate, with large dark brown spot under stigma; hind wing infuscate.

*Head*. From above, 1.4 × wider than long, shiny; lower interocular distance 1.5 × eye height; malar space 0.3 × eye height; occipital margin straight; temple, from above, rounded, slightly longer than eye length; occipital carina 0.1 × diameter of an ocellus; POL:OOL=1.1; malar area densely and finely punctate, remainder of head largely smooth with sparse and fine punctures; A3 3.4 × longer than wide; A4 5.8 × longer than wide, and 1.8 × longer than A3; A5 5.3 × longer than wide, and 1.6 × longer than A3.

*Mesosoma*. Pronotum coarsely areolate-rugose, with one anterior short tooth-like process on lateroventral margin; propleuron dull, mostly finely rugose, posteroventral corner smooth with sparse fine punctures; mesoscutum transverse-carinate anteriorly, irregularly rugose posterior to notauli, anterior part slightly emarginate medially, rounded laterally; notauli deep and wide; scutellum transverse-carinate in middle, irregularly rugose on anterior and posterior margin; axillae, metanotum and propodeum coarsely areolate-rugose; mesopleuron mostly coarsely areolate-rugose with small rugose area anteriodorsally; metapleuron coarsely areolate-rugose; forewing with vein 2-rs+m short, cells SM2 and D1 slightly separated; hind wing with veins somewhat distinct, cells Cu and R1+Rs contiguous; hind coxa rugose; hind basitarsus 11.4 × longer than wide, and 1.1 × of tarsomeres 2–5; pretarsal claw with four tooth-like processes.

*Metasoma*. Smooth, shining, with fine white pubescence on segment 2 to apex; petiole elongate, slender, 2.9 × longer than wide; ovipositor 0.9 × forewing length.

Male. Unknown.

##### Distribution.

China (Zhejiang, Fujian, Hunan).

##### Biology.

Collected in April and August. Host not known.

## Supplementary Material

XML Treatment for Aulacus

XML Treatment for Aulacus flavigenis

XML Treatment for Aulacus magnus

XML Treatment for Aulacus schoenitzeri

XML Treatment for Aulacus sinensis

XML Treatment for Aulacus striatus

XML Treatment for Pristaulacus

XML Treatment for Pristaulacus albitarsatus

XML Treatment for Pristaulacus asiaticus

XML Treatment for Pristaulacus calidus

XML Treatment for Pristaulacus centralis

XML Treatment for Pristaulacus comptipennis

XML Treatment for Pristaulacus excisus

XML Treatment for Pristaulacus fopingi

XML Treatment for Pristaulacus intermedius

XML Treatment for Pristaulacus iosephi

XML Treatment for Pristaulacus karinulus

XML Treatment for Pristaulacus longicornis

XML Treatment for Pristaulacus memnonius

XML Treatment for Pristaulacus nobilei

XML Treatment for Pristaulacus obscurus

XML Treatment for Pristaulacus pieli

XML Treatment for Pristaulacus porcatus

XML Treatment for Pristaulacus pseudoiosephi

XML Treatment for Pristaulacus rufipes

XML Treatment for Pristaulacus rufobalteatus

XML Treatment for Pristaulacus zhejiangensis
